# Exosomal long non-coding RNA TRPM2-AS promotes angiogenesis in gallbladder cancer through interacting with PABPC1 to activate NOTCH1 signaling pathway

**DOI:** 10.1186/s12943-024-01979-z

**Published:** 2024-03-27

**Authors:** Zhiqiang He, Yuhan Zhong, Parbatraj Regmi, Tianrun Lv, Wenjie Ma, Junke Wang, Fei Liu, Siqi Yang, Yanjie Zhong, Rongxing Zhou, Yanwen Jin, Nansheng Cheng, Yujun Shi, Haijie Hu, Fuyu Li

**Affiliations:** 1 Division of Biliary Surgery, Department of General Surgery, West China Hospital, Sichuan University, Chengdu, 610041 P. R. China; 2 Research Center for Biliary Diseases, West China Hospital, Sichuan University, Chengdu, 610041 P. R. China; 3 Key Laboratory of Transplant Engineering and Immunology, NHFPC, West China Hospital, Sichuan University, Chengdu, 610041 P. R. China; 4 Laboratory of Pathology, West China Hospital, Sichuan University, Chengdu, 610041 China

**Keywords:** TRPM2-AS, Gallbladder cancer, Angiogenesis, PABPC1, NOTCH1, IGF2BP2, Exosome

## Abstract

**Background:**

Abnormal angiogenesis is crucial for gallbladder cancer (GBC) tumor growth and invasion, highlighting the importance of elucidating the mechanisms underlying this process. LncRNA (long non-coding RNA) is widely involved in the malignancy of GBC. However, conclusive evidence confirming the correlation between lncRNAs and angiogenesis in GBC is lacking.

**Methods:**

LncRNA sequencing was performed to identify the differentially expressed lncRNAs. RT-qPCR, western blot, FISH, and immunofluorescence were used to measure TRPM2-AS and NOTCH1 signaling pathway expression in vitro. Mouse xenograft and lung metastasis models were used to evaluate the biological function of TRPM2-AS during angiogenesis in vivo. EDU, transwell, and tube formation assays were used to detect the angiogenic ability of HUVECs. RIP, RAP, RNA pull-down, dual-luciferase reporter system, and mass spectrometry were used to confirm the interaction between TRPM2-AS, IGF2BP2, NUMB, and PABPC1.

**Results:**

TRPM2-AS was upregulated in GBC tissues and was closely related to angiogenesis and poor prognosis in patients with GBC. The high expression level and stability of TRPM2-AS benefited from m^6^A modification, which is recognized by IGF2BP2. In terms of exerting pro-angiogenic effects, TRPM2-AS loaded with exosomes transported from GBC cells to HUVECs enhanced PABPC1-mediated NUMB expression inhibition, ultimately promoting the activation of the NOTCH1 signaling pathway. PABPC1 inhibited NUMB mRNA expression through interacting with AGO2 and promoted miR-31-5p and miR-146a-5p-mediated the degradation of NUMB mRNA. The NOTCH signaling pathway inhibitor DAPT inhibited GBC tumor angiogenesis, and TRPM2-AS knockdown enhanced this effect.

**Conclusions:**

TRPM2-AS is a novel and promising biomarker for GBC angiogenesis that promotes angiogenesis by facilitating the activation of the NOTCH1 signaling pathway. Targeting TRPM2-AS opens further opportunities for future GBC treatments.

**Supplementary Information:**

The online version contains supplementary material available at 10.1186/s12943-024-01979-z.

## Background

Gallbladder cancer (GBC) is a common malignant tumor of the biliary tract with a high mortality rate, accounting for 80–95% of all biliary tract tumors [1]. GBC has a strong propensity to invade and metastasize in various ways, including lymph node, liver, vascular, and neural metastasis [2]. The 5-year survival rate for patients with GBC is only 5%, with survival often lasting no longer than six months [1]. The most common clinical adjuvant therapy for GBC is chemotherapy based on gemcitabine and 5-fluorouracil; however, its efficacy is unsatisfactory [3]. Complete surgical resection remains the main treatment for GBC; however, the results of surgical treatment do not meet expectations because patients with GBC are often diagnosed at an advanced stage and are prone to metastasis, with a high recurrence rate and multiple complications [2, 3]. Therefore, the search for novel and effective molecular targets for GBC has prospective therapeutic implications.

Abnormal angiogenesis is a typical feature of hypoxia-induced malignant tumors [4]. Subsequently, the expression of angiogenic factors, such as vascular endothelial growth factor (VEGF), vascular endothelial growth factor receptor (VEGFR), and angiopoietin increases, inducing endothelial cell migration and proliferation, and angiogenic factors continue to stimulate tumor angiogenesis [4]. Compared to the vasculature of normal tissues, the vasculature of tumor tissues shows a chaotic and tangled pattern, with immature and poorly perfused vessels. Dysfunctional vasculature and hypoxic environments contribute to the metastatic spread of tumor cells and resistance to clearance by immune cells [5]. Rapid tumor growth and metastasis depend on novel vessels to provide adequate nutrient and oxygen supply and metabolic waste removal. Deprivation of the vascular supply leads to tumor necroptosis and apoptosis [6]. Therefore, targeting tumor angiogenesis is expected to be a new option for tumor suppression, and based on this, a variety of anti-angiogenic tumor therapeutics have been developed, such as bevacizumab, which specifically targets VEGF/VEGFR, and the multi-targeted agents sorafenib and sunitinib [7]. In-depth mechanistic studies revealed that Fork head box M1 (FoxM1) initiates the transcription of VEGF, thereby activating its expression to promote angiogenesis in GBC [8]. Further evidence has suggested that fibrinogen induces increased expression of intercellular adhesion molecule 1 (ICAM1), promotes GBC angiogenesis, and increases vascular endothelial permeability [9]. Angiogenesis in GBC is also strongly influenced by changes in the tumor microenvironment. Tγδ17 cells can induce GBC cells to secrete IL17, which further induced the production of angiogenic factors, thereby promoting angiogenesis in GBC tumors [10]. However, the lack of research into the concrete mechanisms of angiogenesis in GBC has resulted in a lack of specific drugs to target angiogenesis in GBC. Resistance to conventional drugs and the limitations of intrinsic therapies still severely limit the survival of patients with GBC.

LncRNA is a class of RNA transcripts > 200 nt in length produced by the transcription of genes that do not function as coding proteins [11]. Although lncRNAs do not function as coding proteins, they have been shown to play crucial roles in many physiological and pathological processes. As lncRNAs are capable of interacting with DNA, RNA, and proteins, they function as regulators of gene expression at the transcriptional level, post-transcriptional level, and chromosome remodeling [11, 12]. By affecting the expression of different genes, lncRNA is involved in the regulation of a variety of cellular signaling pathways, including the nuclear factor-κB (NF-κB), Wnt/β-catenin, and NOTCH1 signaling pathways, which are closely associated with cancer [13, 14]. Thus, lncRNAs have a clear effect on tumor development; however, this effect is specific to cancer type and tissue. When lncRNAs act as sponges to compete for miRNAs that bind angiogenesis-associated proteins, they have a significant impact on tumor angiogenesis [15]. Although lncRNAs do not encode proteins, they have been shown to encode micropeptides through which they exert oncogenic effects. LncRNA MLLT4-AS1 encoded a micropeptide, XBP1SBM, which promoted triple-negative breast cancer (TNBC) tumor angiogenesis by upregulating VEGF expression [16]. TRPM2-AS, the antisense form of TRPM2, is a lncRNA with a length of 875 nt. TRPM2-AS drives the progression of various tumors [12, 17]. By acting as competing endogenous RNA for binding to microRNAs, lncRNAs prevent the degradation of miRNAs on their target molecules, and TRPM2-AS promotes the malignant progression of a variety of tumors, including gastric cancer and many other cancers [17]. In addition, the oncogenic effects of TRPM2-AS are also realized through its regulation of gene expression. In colorectal cancer, TRPM2-AS recruited TATA-box-binding protein-associated factor 15 (TAF15) to maintain the stability of its neighboring TRPM2 mRNA, thereby increasing its expression. Regulation of neighboring oncogenes by TRPM2-AS promotes the proliferation of colorectal cancer cells [12]. Although TRPM2-AS has been shown to promote carcinogenesis through various strategies, the link between TRPM2-AS and tumor angiogenesis, particularly in GBC tumors, remains unknown.

This research was intrigued by the function of lncRNA TRPM2-AS in GBC tumor angiogenesis. Here, we found that TRPM2-AS facilitated tumor angiogenesis in GBC both in vitro and in vivo. The stability of TRPM2-AS in GBC cells depends on the m6A modification recognized by insulin like growth factor 2 mRNA binding protein 2 (IGF2BP2). In addition, exosomes favor the transport of TRPM2-AS from GBC cells to HUVECs to exert their pro-angiogenic function. Further mechanistic exploration revealed that the NOTCH1 signaling pathway acts as a crucial downstream pathway for TRPM2-AS. TRPM2-AS enhanced Poly(A) binding protein cytoplasmic 1 (PABPC1)-regulated NUMB degradation, thereby promoting activation of the NOTCH1 signaling pathway. This finding provides a unique insight and shows that lncRNA TRPM2-AS is a novel marker and noteworthy molecule in GBC tumor angiogenesis.

## Materials and methods

### Human tissue samples

Sixty fresh resected GBC tissues and adjacent normal tissues were obtained from the patients admitted to the Department of Hepatobiliary Surgery of the West China Hospital, Sichuan University. In addition, pathological tissues from 12 patients with GBC were obtained from the Pathology Department of West China Hospital. The samples were collected during February 2019 to December 2021. Informed consent was signed for sample collection by all patients, and the study protocol was approved by the Ethics Review Committee of West China Hospital. Demographics, pathology reports, and postoperative oncological outcomes of the patients were collected. Follow-up was conducted until 30 December 2022.

### Cell culture and transfection

Human GBC cell lines GBC-SD, NOZ, SGC-996, EH-GB1, human umbilical vein endothelial cells (HUVECs) and human gallbladder epithelial immortalized cells (HGEICs) were obtained from the Cell Bank of the Chinese Academy of Sciences (Shanghai, China). GBC-SD, NOZ, EH-GB1, HUVECs and HGEICs were cultured in Dulbecco’s modified Eagle’s medium (DMEM), and the SGC-996 cells were cultured in Rosewell Park Memorial Institute (RPMI) 1640 medium (Gibco, USA), supplemented with 10% fetal bovine serum (FBS) (Gibco, USA) and 1% 100 U/mL penicillin and 100 μg/mL streptomycin (Hyclone, USA). Cells were cultured in an incubator with 37 °C, 5% CO_2_.

All the plasmids, viruses, and shRNAs were constructed by TSINGKE (Beijing, China) (Overexpression vector: pLVX-IRES-Puro; knockdown vector: pLKO.1-puro). miRNA mimics were synthesized by RIBOBIO (Guangzhou, China). All transfection assays were performed using Lipofectamine™ 3000 reagent (ThermoFisher Scientific, USA) according to the manufacturer’s instructions. The shRNA sequences are listed in Table S1, and the sequences of all miRNAs are listed in Table S2.

### Exosome extraction and characterization

Exosomes were obtained from the conditioned media derived from GBC cells using an Exosome Extraction Kit (Umibio, China). The morphology of the isolated exosomes was examined using a transmission electron microscopy (FEI, USA). Extracellular vesicle-associated protein markers (CD81, CD9, and CD63) were identified in the isolated exosomes using western blot. Nanoparticle tracking analysis (ParticleMetrix, Germany) was performed to verify the size and concentration of isolated exosomes.

### Co-culture and uptake assays

For the co-culture assay, 5000 HUVECs per well were seeded in a 96-well plate, and PKH26 (Sigma, Germany)-labeled exosomes were added to the cell supernatant. After incubation for 24 h, cells were fixed with 4% paraformaldehyde and permeabilized with 0.5% TritonX-100. Nuclei were stained with DAPI. Cell translocation of exosomes was observed using an inverted fluorescence microscope (Olympus, Japan).

### Proliferation, migration and tube formation assay

The EDU (5-ethynyl-2’-deoxyuridine) assay was performed to assess the cell proliferation of HUVECs according to the manufacturer’s protocol of the kFluor555 Click-iT EDU Imaging Test Kit (KeyGEN BioTECH, China). Briefly, 20000 treated HUVECs were seeded per well in a 96-well plate. EDU solution was added to each well to label the proliferating cells. Two hours later, the cells were fixed and permeabilized, and the EDU-labeled cells were stained with Click-iT reaction mixture. Nuclei were stained with 1 × Hoechst 33342. Images were captured using an inverted fluorescence microscope (Olympus, Japan), and the percentage of EDU-labeled HUVECs in the wells was calculated using ImageJ software (v2021.8.0).

To evaluate the migratory ability of HUVECs, 10000~30000 treated cells suspended in serum-free DMEM were seeded in the upper chamber, and the lower chamber was filled with 600 μL DMEM containing 10% FBS. After 24 h, the cells outside the lower chamber were fixed with 4% paraformaldehyde and stained with 0.1% crystal violet. Representative images were obtained using an inverted fluorescence microscope (Olympus, Japan) and cell numbers were calculated using ImageJ (v2021.8.0).

 Tube formation assay was performed to assess the angiogenic capacity of HUVECs in vitro. Briefly, 20 μL of Matrigel (Corning, USA) per well was added to a 96-well plate and incubated at 37 °C for 1 h. Then, 20000 HUVECs were added to each well and the plate was incubated in an incubator for 6 h. Images of tube-like structures were taken under a light microscope (Olympus, Japan) and counted using ImageJ (v2021.8.0).

### Co-immunoprecipitation (Co-IP)

The Co-IP assay in HUVECs was conducted according to the instructions of the Pierce™ Cross-linked Magnetic Bead Immunoprecipitation/Co-immunoprecipitation Kit (Thermo Fisher Scientific, USA). First, the primary and IgG antibodies were cross-linked to the protein A/G magnetic beads, and then 1 × 10^7^ HUVECs were lysed using IP lysis buffer. Next, the cell lysate was incubated with the cross-linked antibody magnetic beads at 25 °C for 1 h. After washing the antigen- and antibody-bound magnetic beads twice, the bound proteins were extracted and identified using western blot.

### RNA pull-down

The RNA pull-down assay was performed using a RNA pull-down kit (BersinBio, China). Briefly, biotin-labeled TRPM2-AS full-length was synthesized (BersinBio, China) and combined with streptavidin labeled magnetic beads. Next, total proteins of HUVECs with the nucleic acids removed were added, and the mixture was incubated at 25 °C for 2 h. Magnetic beads were collected using a magnetic holder and washed twice. The eluent was used to collect proteins bound to the magnetic beads. The collected proteins were identified by western blot and mass spectrometry.

For the S1m precipitation assay, overexpressed full-length TRPM2-AS plasmid and mutant plasmids 1 (site 271 from A to T), 2 (site 462 from A to T), 3 (site 817 from A to T) with S1m tags were transfected into GBC cells. The cells were then lysed and incubated with streptavidin beads. Finally, the pull-down proteins were eluted using wash buffer and identified by western blot.

### RNA antisense purification (RAP)

Biotin-labeled DNA probes targeting TRPM2-AS were synthesized by TSINGKE (Beijing, China), and experiments were conducted using a RAP kit (BersinBio, China) according to the manufacturer’s instructions. The treated HUVECs were then cross-linked and cleaved to remove DNA. The biotin-labeled probe was incubated with and bound to the streptavidin magnetic beads, and the cell lysate was incubated with the magnetic beads bound to the DNA probe for 1 h. Finally, the RNA and proteins bound to the probe were purified and detected by RT-qPCR and western blot.

### RNA immunoprecipitation (RIP)

The RIP assay was performed using a RIP kit according to the manufacturer’s protocol (BersinBio, China). The cells were lysed to remove DNA, and the remaining lysate was divided into input, IgG, and IP groups. A total of 5 μg of IgG and primary antibody was added to the IgG and IP groups respectively and incubated overnight at 4 °C. The next day, 20 µL protein A/G magnetic beads were added to the IgG group and IP group respectively and incubated again at 4 °C for 1 h. The magnetic beads were collected using a magnetic holder and washed twice. Finally, the RNA protein conjugated to the magnetic beads was eluted. A phenol–chloroform-isoamyl alcohol mixture was used for RNA extraction and purification. RT-qPCR was used to detect the expression levels of the target genes.

### Methylated RNA immunoprecipitation (MeRIP)

The MeRIP assay was performed on GBC-SD and NOZ cells using a MeRIP kit (BersinBio, China). Briefly, total RNA was extracted and fragmented using non-contact ultrasound and then, divided into input, IgG, and IP groups. A total of 5 μg of anti-rabbit IgG and N6-methyladenosine (m^6^A)-specific antibody were added to the IgG and IP groups respectively, and the mixture was incubated at 4 ℃ overnight. The next day, 20 μL of protein A/G magnetic beads were added to the IgG and IP groups respectively and then the two groups were incubated at 4 ℃ for 2 h. Magnetic beads were collected using a magnetic holder and washed three times. The RNA fragments bound to the protein A/G beads were extracted with 200 μL elution buffer. RT-qPCR was performed to analyze the m^6^A-enriched content of the target RNA. The specific primers designed for the m^6^A methylation sites of TRPM2-AS are listed in Table S3.

### Immunohistochemistry (IHC)

Fresh tissues were fixed, embedded in paraffin, and sectioned. The tissue slices were dewaxed with xylene and hydrated using a graded series of decreasing ethanol concentrations. The slides were then placed in Tris–EDTA solution (pH 8.0) and boiled for 10 min to expose tissue antigens. After treatment with 0.3% hydrogen peroxide for 10 min to inactivate endogenous peroxidase in the tissue and blocking with goat serum for 1 h, the tissue slices were incubated with the primary antibody solution overnight. The next day, the tissue slices were incubated with an HRP-conjugated secondary antibody at 25 °C for 1 h and then stained with DAB solution (Cell Signaling, USA). After hematoxylin nucleation, the tissue was dehydrated and sealed with a neutral resin. The tissues were scanned using an inverted fluorescence microscope (Olympus, Japan). ImageJ (v2021.8.0) was used to calculate the microvascular density. For each slice, five representative visual fields were selected and calculated the average of micro vessels number in each field. The microvascular density was shown as the number of micro vessels per square centimeter.

### Luciferase reporter assay

All plasmids used in the luciferase reporter assay were constructed by Tsingke Biotechnology (Beijing, China). To verify the binding of IGF2BP2 to TRPM2-AS, a 50 bp TRPM2-AS fragment containing m^6^A methylation sites was synthesized and cloned into a pGL3 luciferase reporter vector. To detect the binding ability of miR-31-5p and miR-146a-5p to NUMB 3 'UTR, the fragment of NUMB 3' UTR, which was predicted to be targeted by miR-31-5p or miR-146a-5p were cloned into pGL3 control vectors, respectively. After transfection of the plasmid into the indicated cells, luciferase activity was measured using a luciferase detection reagent Luc-Pair Duo-Luciferase HS Assay Kit (GeneCopoeia, USA) according to the manufacturer’s instructions. Cells were lysed, renilla fluorescein substrate and firefly luciferase substrate detection reagents were added to each well, and luminescence intensity was detected using a fluorescence microplate reader (BioTek, USA). Firefly luciferase activity was normalized to that of renilla fluorescein.

### Immunofluorescence (IF)

HUVECs (5 × 10^4^) were implanted and cultured overnight on coverslips. The next day, cells were treated with 4% paraformaldehyde and 0.5% TritonX-100 for fixation and permeabilization. The cells were, then, blocked with goat serum at 25 °C for 1 h, followed by incubation with NOTCH1 intracellular domain (N1ICD) antibody at 4 ℃ overnight. On the second day, the cells were washed thrice with PBS and incubated with the corresponding fluorescent secondary antibody at 25 °C for 1 h. After repeated washes with PBS, DAPI solution was used to stain the nuclei. Representative images were captured using an inverted fluorescence microscope (Olympus, Japan).

### RNA extraction, reverse transcription and quantitative real-time PCR (RT-qPCR)

To exact cellular or exosomal RNA, 1 ml Trizol (Invitrogen, CA, USA) was added to lyse the collected cells or exosomes, followed by the addition of 200 μL chloroform. After centrifugation at 12000 g for 10 min, the supernatant was transferred to another centrifuge tube, and an equal volume of isopropyl alcohol was added. The mixture was then centrifuged at 12000 g for 10 min to collect the precipitate. One milliliter of 75% ethanol was added to wash the RNA precipitates twice. Finally, the RNA precipitates were dissolved in diethylpyrocarbonate (DEPC)-treated water.

Reverse transcription was performed using the HiScript II Reverse Transcriptase Kit (Vazyme Biotech, China) to synthesize cDNA. RT-qPCR was conducted using ChamQTM SYBR@ qPCR Master Mix (Vazyme, China) on CFX96TM Touch Real-Time PCR System (Bio-Rad, USA). Relative RNA expression levels were calculated using 2^−ΔΔCt^ method and normalized to GAPDH. The sequences of all primers are listed in Table S3.

### Fluorescent in situ hybridization (FISH)

In situ hybridization was performed using a RiboTM Fluorescent in Situ Hybridization Kit (RIBOBIO, China). After treatment, 5 × 10^4^ HUVECs were seeded on a coverslip in a 24-well plate. The next day, the cells on the coverslip were fixed, permeabilized, and blocked with a pre-hybridization solution, followed by incubation with the hybridization solution containing the TRPM2-AS probe at 37 ℃ overnight. The third day, the cells were washed with 42 ℃ 4 × SSC containing 0.1% Tween20, 2 × SSC, and 1 × SSC solutions and the nuclei were stained with DAPI. HUVECs were observed under an inverted fluorescence microscope (Olympus, Japan).

### Western blot

Total protein was extracted from tissues, cells, or exosomes using RIPA buffer (Beyotime Biotechnology, China). Protein concentration was assessed using a BCA kit (Beyotime Biotechnology, China). The extracted protein samples were subjected to sodium dodecyl sulfate–polyacrylamide gel electrophoresis to separate proteins with different molecular weights. The proteins were then transferred to PVDF membranes (Millipore, USA) at a constant current of 250 mA and blocked with 5% skim milk at 25 °C for 1 h. The membranes were then incubated with corresponding primary antibody at 4 °C overnight. After repeated washing steps, the membranes were further incubated with horseradish peroxidase-conjugated secondary antibodies (HUABIO, China) for 1 h. Finally, chemiluminescence signals were detected using an Enhanced Chemiluminescence Detection Kit (NCM Biotech, China). Images were captured using a gel imaging system (Bio-Rad, USA). The details of the antibodies used are listed in Table S4.

### Animal studies

Animal experiments were conducted in accordance with the guidelines for the care of laboratory animals. All animals were purchased from Beijing HFK Bioscience Company (HFK Bioscience, China) and treated according to the NIH Guide for the Care and Use of Laboratory Animals.

Six-week-old female nude BALB/c mice were used to establish the xenograft tumor model. Each group consisted of five to six mice. Tumor cells (2 × 10^6^) were implanted into the right underarm of the mice. The diameters of the GBC-SD cell-derived subcutaneous tumors were measured using Vernier calipers every 7 days, and the xenograft tumors were removed on day 28. NOZ cell-derived tumors were measured every 5 days, and xenograft tumors were removed on day 20. Xenograft tumors were weighed using an analytical balance, and the volume was estimated using the formula 0.5 × length × width^2^.

Six-week-old female nude BALB/c mice were used to establish the lung metastasis model. Each group consisted of five mice. 2 × 10^6^ GBC-SD or NOZ cells were injected into the tail vein, the nude mice were euthanized, and the lung tissue was removed after 40 days. The number of pulmonary metastatic nodules was calculated by hematoxylin and eosin (H&E) staining, and the microvascular density of the pulmonary metastatic nodules was evaluated using CD34 immunohistochemistry.

### Statistical analyses

Each experiment was repeated at least three times. Statistical analyses were performed using IBM SPSS (version 25), GraphPad Prism (version 9.1), and the R Project for Statistical Computing (version 4.3). Pearson’s correlation coefficient was used to determine the correlation between TRPM2-AS expression and microvascular density. A Pearson’s correlation coefficient (r) greater than 0.5 was considered a strong positive correlation, and less than -0.5 was considered a strong negative correlation. Categorical variables were compared using the chi-squared test. Kaplan–Meier survival analysis with a log-rank test was used to compare survival outcomes. *P* value < 0.05 was considered statistically significant.

## Results

### TRPM2-AS is overexpressed in GBC and positively associated with tumor angiogenesis and poor prognosis

**Fig. 1:**
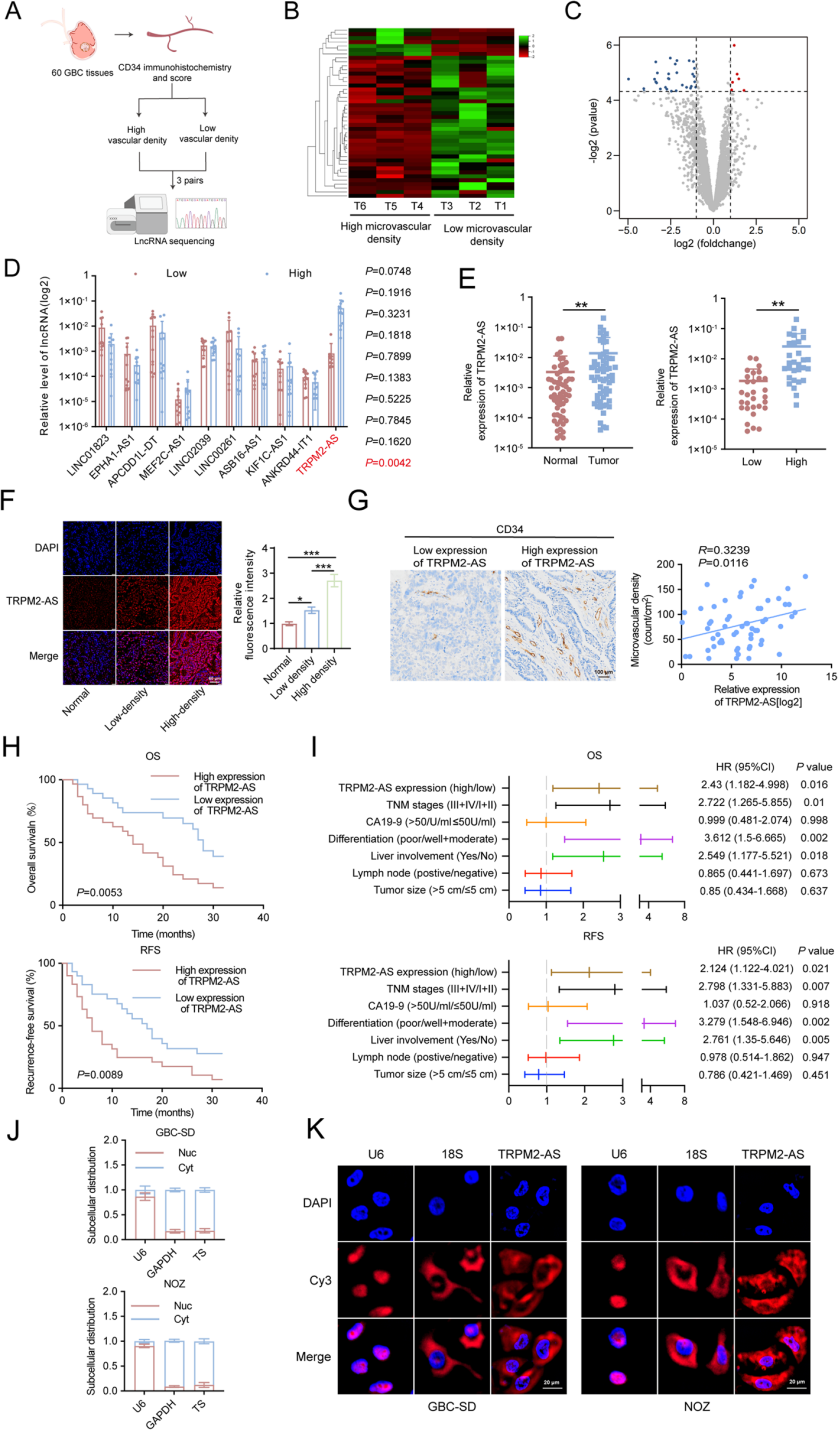
TRPM2-AS is overexpressed in GBC and is associated with high microvascular density and poor prognosis. **A** Schematic of CD34 immunohistochemistry and lncRNA sequencing of GBC tissues. The microvascular density of 60 GBC tissues was evaluated by CD34 immunohistochemistry and three GBC tissues with the lowest microvascular density and three with the highest microvascular density were selected for lncRNA sequencing. **B**, **C** Heatmap (**B**) and volcano plot (**C**) of the differential expression of genes in three GBC tissues with high microvascular density or low microvascular density. **D** RT-qPCR assessment of the top ten lncRNAs with the most significant changes in RNA expression levels. **E** Relative expression of TRPM2-AS in GBC tissues and in adjacent normal tissues (left)/in low-density GBC tissues and in high-density GBC tissues (right) was quantified using RT-qPCR. **F** FISH visualization of TRPM2-AS in normal GBC tissues/low-density GBC tissues/ high-density GBC tissues. Blue fluorescence: DAPI-stained nuclei; red fluorescence: Cy3-labeled TRPM2-AS. Scale bar: 60 μm. **G** Representative images of the immunohistochemical staining of CD34 expression in tissues with high/low TRPM2-AS expression and Pearson’s correlation analysis of the positive correlation between microvascular density and TRPM2-AS expression. Scale bar: 100 μm. **H** Kaplan–Meier curve analysis of the correlation between the expression level of TRPM2-AS and overall survival (OS)/recurrence-free survival (RFS) in GBC patients. **I** Multivariate analysis of the prognostic factors for OS and RFS in GBC patients. **J** Quantification of TRPM2-AS distribution in nuclear and in cytoplasm. **K** Representative images of FISH analysis showing the subcellular localization of TRPM2-AS in GBC-SD and NOZ cells. Blue fluorescence: DAPI-stained nuclei; red fluorescence: Cy3-labeled U6, 18S and TRPM2-AS probes. Scale bar: 20 μm. Data were assessed with unpaired Student’s *t* test or one-way ANOVA and presented as mean ± SD. * *P* < 0.05; ** *P* < 0.01; *** *P* < 0.001

To investigate the mechanism of angiogenesis in GBC, we evaluated the microvascular density of 60 GBC specimens by CD34 immunohistochemical staining and then performed lncRNA sequencing on the three GBC tissues with the highest and lowest vascular density (Fig. 1A). Under the condition of log2|(fold-change) |> 1 and *P* < 0.05, six lncRNAs were upregulated and 37 lncRNAs were downregulated (Fig. 1B, C). Considering the small number of samples used for sequencing, we used RT-qPCR to detect the top ten lncRNAs with the most obvious *P* values in 12 pairs of low-density vascular GBC tissues and high-density vascular GBC tissues. The results showed that only TRPM2-AS was significantly upregulated in GBC tissues with high microvascular density, whereas the other lncRNAs exhibited no statistical differences (Fig. 1D). We first examined the expression level of TRPM2-AS in human gallbladder epithelial immortalized cells (HGEICs) and in several common GBC cells (GBC-SD/996/NOZ/EH-GB1), respectively. We observed that TRPM2-AS was obviously more highly expressed in GBC cells than in normal gallbladder cells (Fig. S1A). Further RT-qPCR and FISH experiments showed that TRPM2-AS was upregulated in tumor tissues compared to matched normal tissues, and that TRPM2-AS was significantly higher in GBC tissues with high microvascular density than in GBC tissues with low microvascular density (Fig. 1E, F). Linear regression analysis also confirmed that TRPM2-AS expression was positively correlated with microvascular density in 60 GBC samples (Fig. 1G). In addition, by analyzing the clinical-pathophysiological characteristics of GBC patients with high and low TRPM2-AS expression, we found that patients with high TRPM2-AS expression tended to have a larger tumor size, higher CA19-9 levels, higher rates of lymph node metastasis, liver invasion, lower tumor differentiation, and later tumor node metastasis (TNM) stages (Table S5). Kaplan–Meier analysis revealed an association between TRPM2-AS and reduced overall survival (OS) and recurrence-free survival (RFS) in patients with GBC (Fig. 1H). Through univariate and multivariate Cox regression analyses, we found that the TRPM2-AS expression level was an independent prognostic factor in patients with GBC (Fig. 1I, Tables S6 and S7). Therefore, these results suggest that TRPM2-AS is highly expressed in GBC tissues and is closely correlated with the microvascular density of GBC tissues and the poor prognosis of GBC patients. Furthermore, we confirmed the subcellular localization of TRPM2-AS by nuclear isolation and FISH assays, which showed that TRPM2-AS was mainly localized to the cytoplasm of GBC cells (Fig. 1J, K).

### TRPM2-AS promotes tumor angiogenesis in vivo

**Fig. 2:**
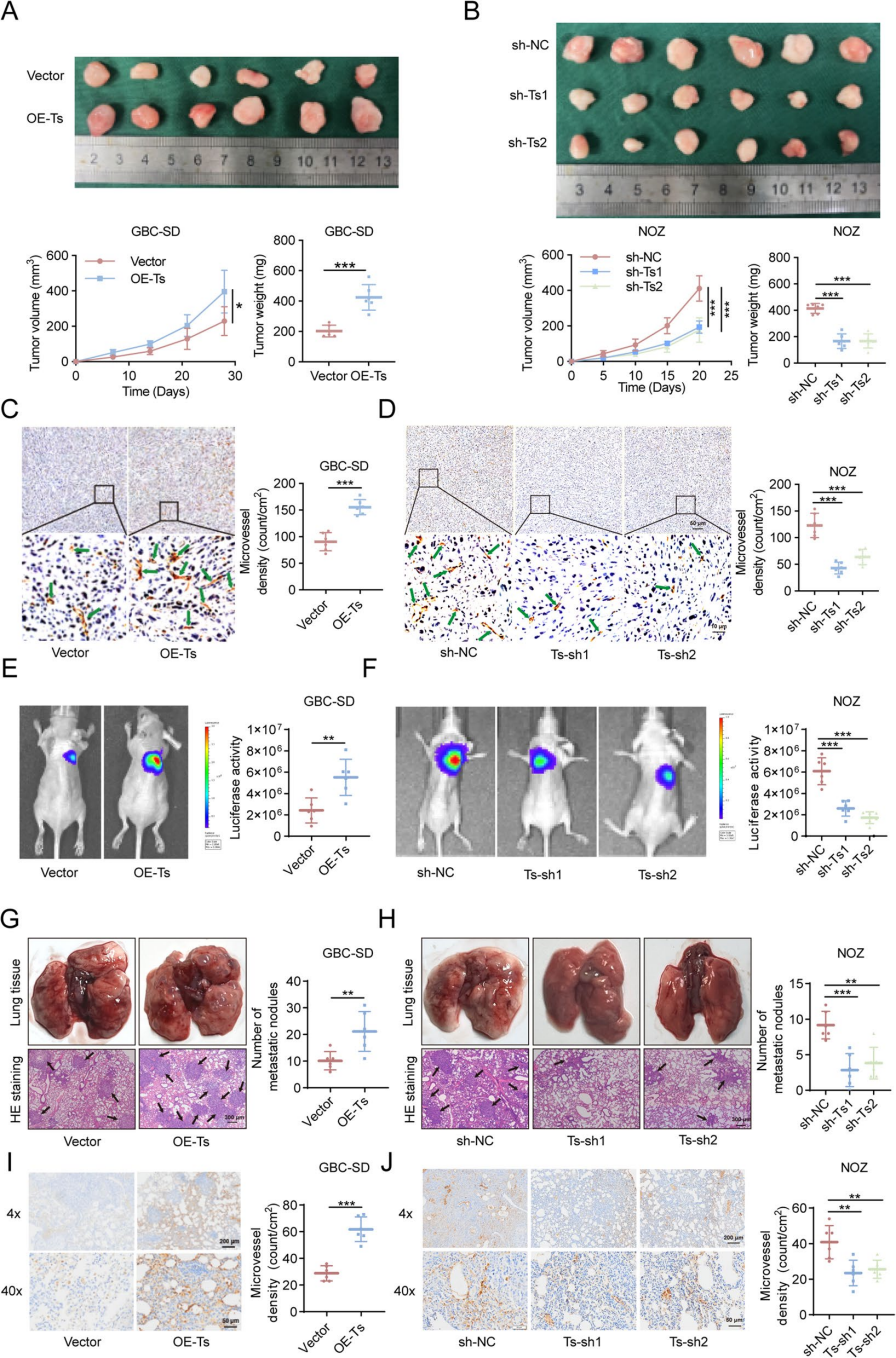
TRPM2-AS promotes GBC angiogenesis in vivo. **A**, **B** Representative images and quantification of surgically removed GBC tumors at day 28 in the TRPM2-AS overexpression group (**A**) and at day 20 in the TRPM2-AS knockdown group (**B**) (*n* = 6). **C**, **D** Microvascular density of GBC tumor sections in the TRPM2-AS overexpression (**C**) and knockdown (**D**) groups was evaluated by CD34 immunohistochemical staining. Scale bar: 10/50 μm. **E**, **F** Representative images of bioluminescence imaging at day 40 showing the pulmonary metastasis in the TRPM2-AS overexpression (**E**) and knockdown (**F**) groups (*n* = 5). **G**, **H** Representative images of surgically removed lung tissues and H&E-staining showing the pulmonary metastasis in the TRPM2-AS overexpression (**G**) and knockdown (**H**) groups. Scale bar: 300 μm. **I**, **J** Representative immunohistochemical staining images and quantification of dissected lung tissues detecting the microvascular density in the TRPM2-AS overexpression (**I**) and knockdown (**J**) groups. Scale bar: 50/200 μm. Data were assessed with unpaired Student’s *t* test, one-way ANOVA, or two-way ANOVA and presented as mean ± SD. ** *P* < 0.01; *** *P* < 0.001

We were intrigued to asked whether TRPM2-AS had pro-angiogenesis effects on GBC. We generated GBC cell lines with TRPM2-AS stable overexpression and knockdown (Fig. S1B, C). The effects of TRPM2-AS overexpression and knockdown on GBC angiogenesis were verified in xenograft tumor and lung metastasis models. Heterotopic inoculation of TRPM2-AS overexpressing GBC cells resulted in a faster rate of tumor growth and significantly increased endpoint tumor weight and volume (Fig. 2A), as well as the microvascular density of resected tumors (Fig. 2C). However, when TRPM2-AS was knocked down, these indices were reversed (Fig. 2B, D). In addition, we obtained similar results in the lung metastasis model; luciferase activity in metastatic tumors (Fig. 2E), the number of pulmonary metastatic nodules (Fig. 2G), and the microvascular density of the nodules (Fig. 2I) were all remarkably increased in the TRPM2-AS overexpression group, whereas in the TRPM2-AS knockdown group, the advantages of luciferase activity, pulmonary metastasis, and increased nodule vascular density were abolished (Fig. 2F, H, J).

### IGF2BP2 increases the stability of TRPM2-AS and promotes its expression in an m^6^A-dependent manner

**Fig. 3:**
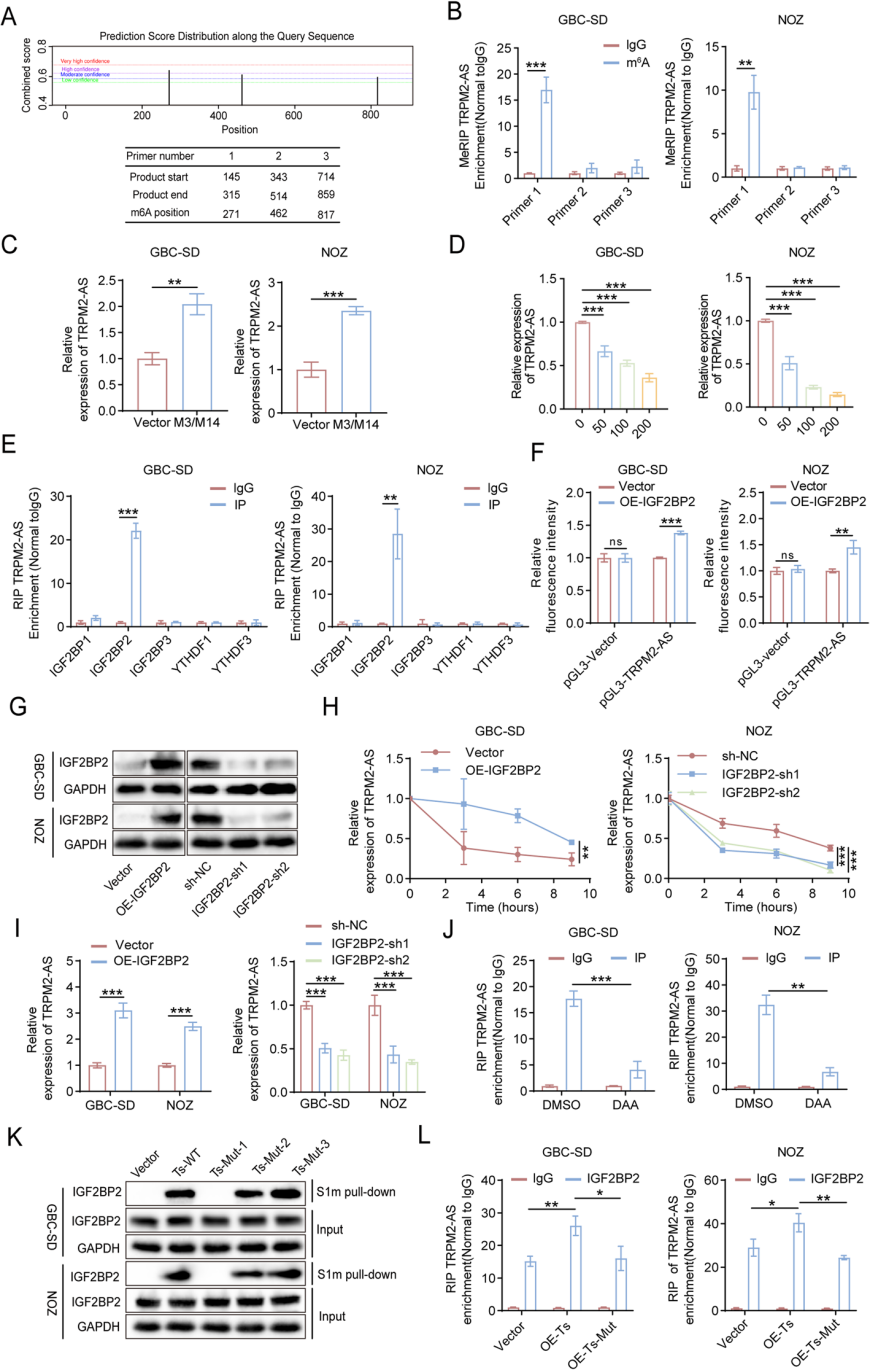
IGF2BP2-mediated m^6^A modification favors the stability of TRPM2-AS. **A** m^6^A modification sites of TRPM2-AS were predicted using SRAMP (http://www.cuilab.cn/sramp). **B** MeRIP and RT-qPCR assays revealing the m^6^A enrichment of TRPM2-AS by using three different primers. **C** The relative expression levels of TRPM2-AS in GBC cells were measured by RT-qPCR with/without METTL3/METTL14 overexpression. **D** RT-qPCR showing the decreased expression of TRPM2-AS upon treatment with 0, 50, 100, 200 μM DAA. **E** RIP analysis revealing the binding levels of TRPM2-AS with IGF2BP1, IGF2BP2, IGF2BP3, YTHDF1and YTHDF3. **F** The binding of TRPM2-AS to IGF2BP2 was identified using a dual-luciferase reporter system. **G** Western blot validation of IGF2BP2 protein expression levels in IGF2BP2 stable overexpression/knockdown GBC cells by lentivirus transfection. **H** Relative expression level of TRPM2-AS in IGF2BP2 overexpression/knockdown GBC-SD and NOZ cells after treated with actinomycin for different time point. **I** RT-qPCR showing the relative expression level of TRPM2-AS with IGF2BP2 overexpression/knockdown. **J** RIP analysis revealing the binding of TRPM2-AS and IGF2BP2 after the m^6^A methylation of TRPM2-AS was inhibited by DAA. **K** S1m pull-down assessment showing the changes in IGF2BP2 level with three different TRPM2-AS m^6^A site mutations. **L** RIP analysis verifying the enrichment of TRPM2-AS in the control group and in the IGF2BP2 overexpression group with transfection of TRPM2-AS or TRPM2-AS m^6^A sites mutation (Mut-1) overexpression plasmid. Data were assessed with unpaired Student’s *t* test or one-way ANOVA and presented as mean ± SD. * *P* < 0.05; ** *P* < 0.01; *** *P* < 0.001

m^6^A methylation is the most common RNA modification that plays a critical role in RNA splicing, translation, and stability [18]. Therefore, we used SRAMP (http://www.cuilab.cn/sramp) to predict the presence of m^6^A methylation sites in TRPM2-AS and identified three possible m^6^A methylation sites. For further confirmation, we designed three pairs of primers specific for these three sites (Fig. 3A). MeRIP assays showed that TRPM2-AS was significantly enriched in the primer 1 group, indicating 271 sites where m^6^A methylation occurred in TRPM2-AS (Fig. 3B). To explore the role of m^6^A methylation in TRPM2-AS expression in GBC cells, we overexpressed M3/M14 (Mettle 3/Mettle 14) in GBC cells and found that both m^6^A methylation (Fig. S2A) and TRPM2-AS expression levels (Fig. 3C) were substantially elevated. Next, we used deazadenosine (DAA) to inhibit methyltransferase activity. After DAA treatment, the m^6^A methylation level of TRPM2-AS decreased (Fig. S2B), and TRPM2-AS expression decreased in a DAA dose-dependent manner (Fig. 3D). Above experiments demonstrated that increased m^6^A modification of TRPM2-AS could increase the expression of TRPM2-AS. The effect of m^6^A methylation on RNA was largely depended on the m^6^A reader. Based on the function of m^6^A readers, only IGF2BP1, IGF2BP2, IGF2BP3, YTH N6-methyladenosine RNA-binding protein 1 (YTHDF1), and YTH N6-methyladenosine RNA-binding protein 3 (YTHDF3) were able to promote RNA expression by recognizing m^6^A methylation. To identify the m^6^A reader that mediates the m^6^A modification of TRPM2-AS, we performed an RIP assay using the corresponding antibodies of the above five m^6^A readers and then used RT-qPCR to detect the TRPM2-AS content in the pull-down RNA. RT-qPCR results showed that among these readers, TRPM2-AS was highly enriched only in the IGF2BP2 group (Fig. 3E). As expected, the dual-luciferase reporter assay showed that IGF2BP2 directly interacted with TRPM2-AS (Fig. 3F). To investigate the effect of IGF2BP2 expression on the stability and expression of TRPM2-AS, we constructed IGF2BP2 overexpression and knockdown GBC cell lines (Fig. 3G). We used actinomycin D to inhibit global RNA synthesis and then used RT-qPCR to detect the TRPM2-AS content in tumor cells at different time points. The results showed that the stability and expression levels of TRPM2-AS increased with IGF2BP2 overexpression and significantly decreased with IGF2BP2 knockdown (Fig. 3H, I). Therefore, IGF2BP2 interacts with TRPM2-AS and promotes the stability of TRPM2-AS. However, whether the promoting effect of IGF2BP2 on TRPM2-AS is dependent on m^6^A modification of TRPM2-AS remains unknown.

To verify that m^6^A modification was the basis of the interaction between IGF2BP2 and TRPM2-AS, we treated GBC cells with DAA to inhibit the m^6^A methylation of TRPM2-AS. The RIP assay revealed that the level of TRPM2-AS enriched by IGF2BP2 was markedly decreased (Fig. 3J), and the significant promotive effect of IGF2BP2 on TRPM2-AS expression was diminished (Fig. S2C), indicating the necessity of m^6^A methylation. Furthermore, we synthesized RNA probes with m^6^A modifications at 271, 462, and 817 sites, which were predicted using the SRAMP website, and conducted an S1m pull-down assay for further determination. We found that when the nucleotide at 271 site was mutated, IGF2BP2 protein levels decreased significantly (Fig. 3K). Therefore, the 271 site of m^6^A modification site was indispensable for the binding of IGF2BP2 to TRPM2-AS. Next, we transfected with TRPM2-AS and mutated TRPM2-AS (TRPM2-AS m^6^A site mutation) overexpression plasmids. Compared with the control group, the RIP results showed that the level of IGF2BP2-bound TRPM2-AS was also strikingly increased by TRPM2-AS overexpression. However, there were no significant changes in the IGF2BP2-bound TRPM2-AS levels when TRPM2-AS-MUT was overexpressed (Fig. 3L). When TRPM2-AS was overexpressed, IGF2BP2 promoted the expression of TRPM2-AS, but when TRPM2-AS-MUT was overexpressed, IGF2BP2 had no effect (Fig. S2D). These results strongly suggest that IGF2BP2 increases the stability of TRPM2-AS by mediating m^6^A modification of TRPM2-AS.

Consistent with the oncogenic and pro-angiogenic role of TRPM2-AS in GBC cells, RT-qPCR (Fig. S3A), western blot (Fig. S3B), and immunohistochemistry (Fig. S3C) also showed that the expression level of IGF2BP2 was increased in tumor tissues, especially in those with high microvascular density. IGF2BP2 was also positively associated with microvascular density (Fig. S3D) and poor patient outcomes (Fig. S3E, F). Univariate and multivariate Cox regression analyses showed that IGF2BP2 was an independent prognostic factor in patients (Fig. S3G, H, Tables S8 and S9).

### Exosomes transfers TRPM2-AS from GBC cells to HUVECs to promote angiogenesis

In the tumor microenvironment, lncRNAs promote tumor angiogenesis in various ways, and the pivotal role of exosomes (involved in lncRNA transfer) has been repeatedly highlighted [19]. To investigate whether exosomes mediated the transfer of TRPM2-AS from GBC cells to HUVECs, we generated TRPM2-AS overexpression GBC-SD, NOZ, SGC-996, and EH-GB1 cells via lentiviral transfection. RT-qPCR results showed that with TRPM2-AS overexpression in GBC cells, both TRPM2-AS expression in exosomes and exosome-treated HUVECs appeared to be upregulated, with NOZ cells showing the most significant upregulation (Fig. S4A, B). Therefore, exosomes produced by NOZ cells were selected for follow-up experiments. We then assessed the level of TRPM2-AS in HUVECs after co-culture HUVECs with TRPM2-containing exosomes for different times, and our RT-qPCR results showed that co-culture for 24 h resulted in the highest level of TRPM2-AS, so we chose this time for subsequent assays (Fig. S4C). The markers CD9, CD63, and CD81 in the exosomes produced by NOZ cells were detected by western blot (Fig. S4D), and the typical goblet morphology of NOZ exosomes was observed by transmission electron microscopy (Fig. S4E). Particle size experiments showed that the extracted exosomes had a diameter between 50–150 nm (Fig. S4F), which was consistent with the diameter of the exosomes. We then investigated the transport of TRPM2-AS from GBC cells to HUVECs by labelling exosomes with PKH26 and co-culturing them with HUVECs. The results showed that the exosomes transported TRPM2-AS to HUVECs (Fig. S4G).

**Fig. 4:**
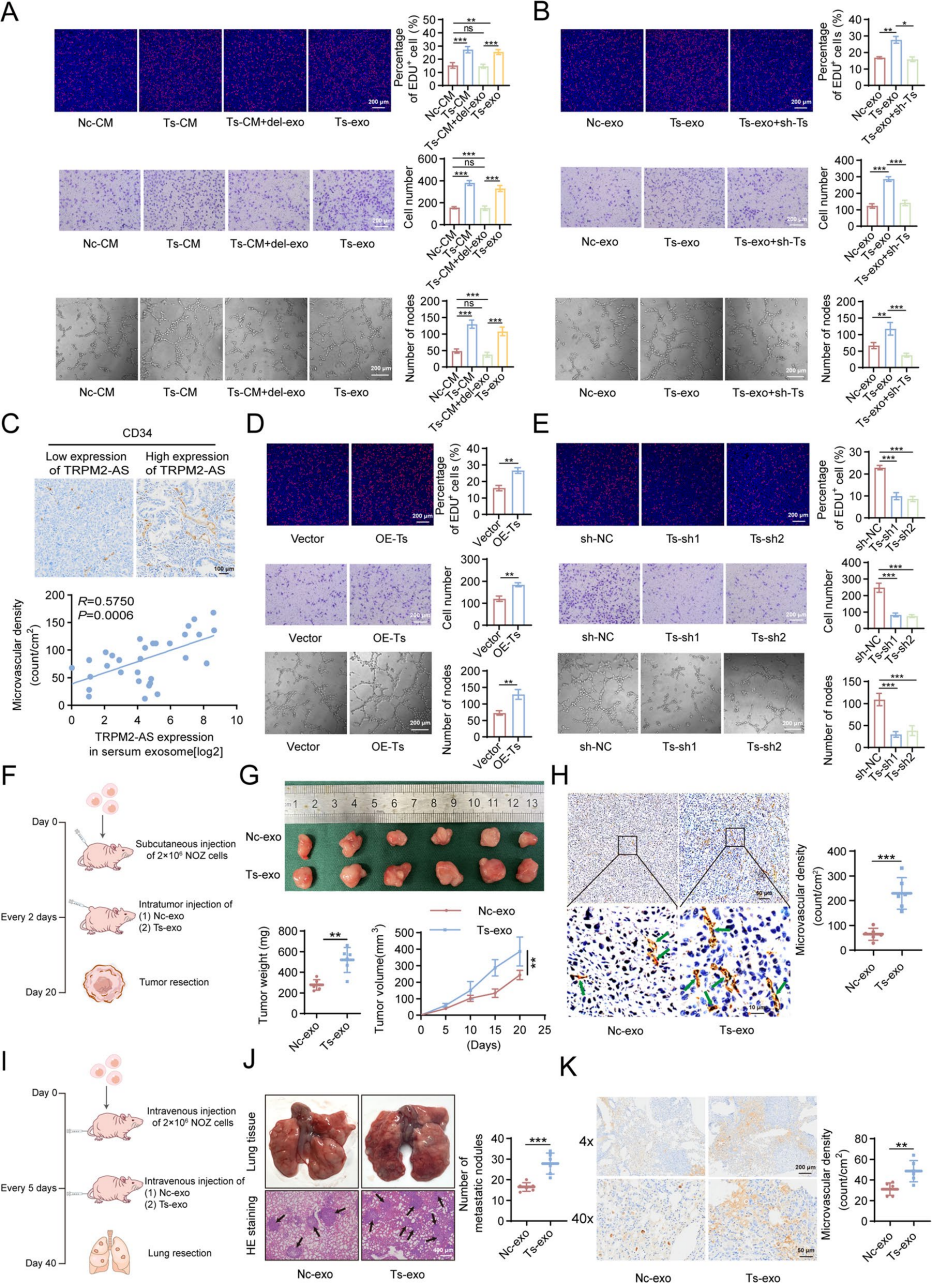
Exosomes transport TRPM2-AS to HUVECs and promote tumor angiogenesis. **A**, **B** EDU, transwell, and tube formation assays verifying the angiogenic ability of HUVECs cultured in Nc-CM/Ts-CM/Ts-CM + del-exo/Ts-exo (**A**), Nc-exo/Ts-exo/Ts-exo + sh-Ts (**B**). Nc-CM: conditioned medium from NOZ cells overexpressing empty plasmid. Ts-CM: conditioned medium from NOZ cells overexpressing TRPM2-AS. Ts-CM + del-exo: culture medium from NOZ cells overexpressing TRPM2-AS after exclusion of exosomes. Ts-exo: exosomes from NOZ cells overexpressing TRPM2-AS. Nc-exo: exosomes from NOZ cells overexpressing empty plasmid. Ts-exo + sh-Ts: knockdown TRPM2-AS using shRNA after HUVECs were co-cultured with Ts-exo. Red fluorescence: EDU-positive cells (EDU^+^). Scale bar: 200 μm (EDU/transwell/tube formation). **C** Representative images of the immunohistochemical staining of CD34 expression in tissues with high/low TRPM2-AS expression in serum exosomes from 32 GBC patients and Pearson’s correlation analysis of the positive correlation between microvascular density of GBC tissues and TRPM2-AS expression in serum exosomes. **D**, **E** EDU, transwell, and tube formation assays verifying the angiogenic capacity of HUVECs with overexpression (**D**) and knockdown (**E**) of TRPM2-AS. **F**-**H** BALB/c nude mice were treated with Nc-exo/Ts-exo every 2 days after subcutaneous injection of 2 × 10^6^ NOZ cells (**F**). Representative images of surgically resected GBC tumour. Tumor weight and volume were quantified on day 20 (**G**) (*n* = 6). Representative images showing the CD34 expression level in surgically removed GBC tumors (**H**). Scale bar: 10 μm. **I**-**K** BALB/c nude mice were intravenously treated with Nc-exo/Ts-exo every 5 days after intravenous injection of 2 × 10^6^ NOZ cells (**I**). Representative image and H&E staining of surgically resected lung (**J**) (*n* = 5). Immunohistochemical staining showing the microvascular density of pulmonary metastatic nodules (**K**). Scale bar: 50/200 μm. Data were assessed with unpaired Student’s *t* test, one-way or two-way ANOVA and presented as mean ± SD. * *P* < 0.05; ** *P* < 0.01; *** *P* < 0.001; ns, no significance

To confirm that exosomes, rather than other mediators, were involved in the pro-angiogenic effects of TRPM2-AS in GBC cells, we co-cultured HUVECs in NOZ cell-conditioned medium (Nc-CM), TRPM2-AS overexpressing NOZ cell-conditioned medium (Ts-CM), TRPM2-AS overexpressing NOZ cell-conditioned medium with exosomes removed (Ts-CM + del-exo), and exosomes extracted from TRPM2-AS overexpressing NOZ cell-conditioned medium (Ts-exo). The results of EDU, transwell, and tube formation assays revealed that the proliferation, migration, and tube formation ability of HUVECs in the Ts-CM and Ts-exo groups were significantly higher than those in the Nc-CM group, while there was no statistical significance in the Ts-CM + del-exo group compared to the Nc-CM group (Fig. 4A). Concomitantly, we also observed that the expression of B-cell chronic lymphocytic leukemia/lymphoma-2 (BCL-2), cyclinD1and vascular endothelial growth factor A (VEGFA), increased in the Ts-CM and Ts-exo groups while the expression of BCL-2-associated X protein (BAX) drastically decreased (Fig. S5A). Therefore, exosomes are the key mediators of TRPM2-AS in promoting angiogenesis.

Tumor exosomes contain many types of signaling molecules such as proteins, mRNA, and non-coding RNA [20]. To confirm that TRPM2-AS, but not other molecules, was loaded into exosomes and delivered to endothelial cells, we co-cultured control exosomes extracted from control NOZ cells (Nc-exo) and Ts-exo with HUVECs and used shRNA to reverse TRPM2-AS overexpression induced by Ts-exo. The cellular and molecular results showed that the proliferation, migration, and angiogenesis of HUVECs were substantially enhanced in the Ts-exo group compared to those in the Nc-exo group, while TRPM2-AS knockdown reversed this effect (Figs. 4B and S5B). We found that the level of TRPM2-AS in exosomes extracted from the serum of 32 patients was positively correlated with the microvascular density of the tumor tissues (Fig. 4C). Therefore, Ts-exo promotes angiogenesis in GBC cells by delivering TRPM2-AS rather than other proteins or RNA molecules.

To verify whether TRPM2-AS has a pro-angiogenic function in endothelial cells, we directly overexpressed and knocked down TRPM2-AS in HUVECs. Indeed, overexpression of TRPM2-AS significantly enhanced the proliferation, migration, and angiogenic ability (Figs. 4D and S5C), while knockdown of TRPM2-AS weakened these abilities (Figs. 4E and S5D).

To test whether exosomal TRPM2-AS promoted angiogenesis in vitro, we subcutaneously injected 2 × 10^6^ NOZ cells into BALB/c nude mice to construct xenograft tumor models. Subsequently, Nc-exo and Ts-exo were injected into the subcutaneous tumors every two days. Twenty days later, the nude mice were sacrificed, and the subcutaneous tumors were harvested (Fig. 4F). Ts-exo treatment resulted in a faster subcutaneous tumor growth rate, and heavier and larger endpoint tumors (Fig. 4G). Regarding microvascular density, CD34 immunohistochemistry showed that the microvascular density in the Ts-exo group was significantly higher than that in the Nc-exo group (Fig. 4H). We constructed a lung metastasis model by injecting 2 × 10^6^ NOZ cells into the tail vein of BALB/c nude mice and intravenously administering Nc-exo and Ts-exo every five days. After 40 days, the mice were sacrificed to evaluate lung metastasis (Fig. 4I). Haematoxylin and eosin staining showed that Ts-exo treatment led to a remarkable difference in lung metastasis, as manifested by a higher number of lung metastases (Fig. 4J). CD34 immunohistochemistry also showed that the microvascular density of the metastatic tumors in the Ts-exo group was significantly higher than that in the Nc-exo group (Fig. 4K).

### TRPM2-AS favors the activation of NOTCH1 signaling pathway, thus promoting angiogenesis

**Fig. 5:**
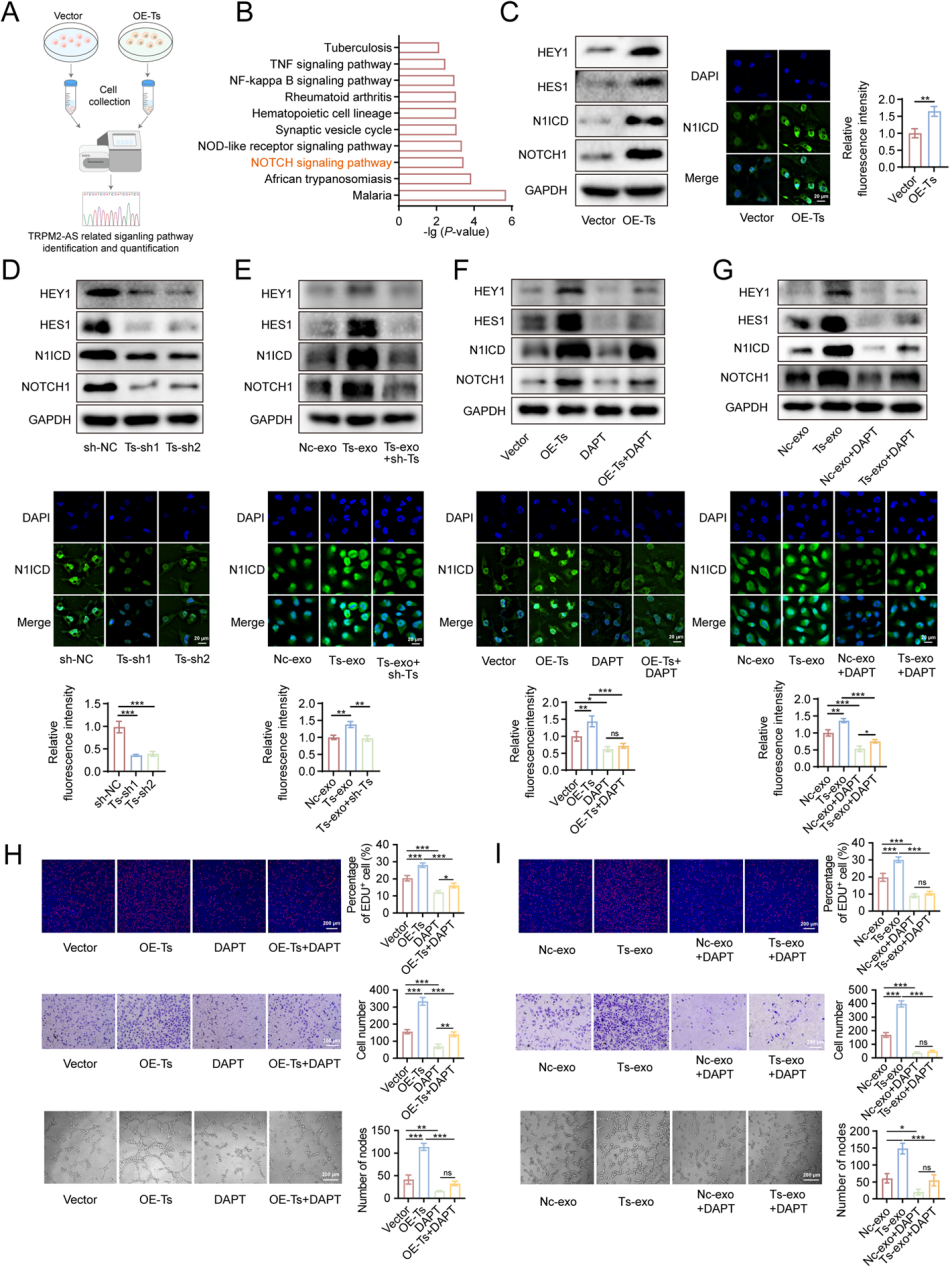
TRPM2-AS promotes the activation of NOTCH1 signaling pathway in HUVECs. **A**, **B** Transcriptomic sequencing was performed on 1 × 10^7^ control and TRPM2-AS overexpressing HUVECs cells, and KEGG was performed to analyse the significantly altered signaling pathways. **C**-**E** Western blot validation of HEY1/HES1/N1ICD/NOTCH1 expression level in TRPM2-AS overexpressing, knockdown, and control HUVECs and immunofluorescence verification of N1ICD expression level in TRPM2-AS overexpressing, knockdown, and control HUVECs and in HUVECs cultured in Nc-exo/Ts-exo with/without knockdown of TRPM2-AS. Green fluorescence: corresponding primary antibody stained N1ICD; blue fluorescence: DAPI-stained nuclei. Scale bar: 20 μm. **F**, **G** Western blot analysis showing the HEY1/HES1/N1ICD/NOTCH1 expression levels and representative immunofluorescence images of N1ICD expression in TRPM2-AS overexpressing/control HUVECs with/without DAPT treatment and in HUVECs cultured in Nc-exo/Ts-exo with/without DAPT treatment. **H**, **I** EDU, transwell, and tube formation assays to detect the angiogenic ability of TRPM2-AS overexpressing/control HUVECs with/without DAPT treatment (**H**), and HUVECs cultured in Nc-exo/Ts-exo with/without DAPT treatment (**I**). Red fluorescence: EDU-positive cells (EDU^+^). Scale bar: 200 μm (EDU/transwell/tube formation). Data were assessed with unpaired Student’s *t* test or one-way ANOVA and presented as mean ± SD. * *P* < 0.05; ** *P* < 0.01; *** *P* < 0.001; ns, no significance

Our results revealed the pro-angiogenic effects of TRPM2-AS on GBC cells, and we sought to explore the mechanism by which TRPM2-AS promoted HUVECs proliferation, migration, and tube formation. Therefore, we performed transcriptomic sequencing of TRPM2-AS overexpressing HUVECs and control HUVECs (Fig. 5A). Kyoto Encyclopedia of Genes and Genomes (KEGG) enrichment analysis showed that among the predicted signaling pathways with which TRPM2-AS interacts, the NOTCH signaling pathway was distinctly activated (Fig. 5B). The NOTCH1 signaling pathway has been demonstrated to regulates and plays an important role in angiogenesis [7]. Subsequently, western blot was used to detect the expression of key molecules in the NOTCH1 signaling pathway, including NOTCH1, N1ICD, HES1, and HEY1. Immunofluorescence was used to detect the fluorescence intensity of N1ICD in the nucleus. The results showed that overexpression of TRPM2-AS significantly increased the expression levels of key molecules in the NOTCH1 signaling pathway, whereas inhibition of TRPM2-AS significantly suppressed their expression (Fig. 5C, D). Consistently, the NOTCH1 signaling pathway was activated in the Ts-exo group compared to the Nc-exo group, whereas TRPM2-AS knockdown showed the opposite results (Fig. 5E).

To further verify the key role of NOTCH1 signaling pathway in TRPM2-AS mediated-proliferation, migration, and tube formation of HUVECs, we used DAPT (N-[N-(3, 5-difluorophenyl)-l-alanyl] -s-phenylglycine t-butyl ester), a known blocker of NOTCH1 signaling pathway, to inhibit the function of γ-secretase and thus to inhibit the activation of NOTCH1 signaling pathway. DAPT treatment significantly reversed the activation of the NOTCH1 signaling pathway induced by TRPM2-AS overexpression and Ts-exo (Fig. 5F, G). Consistent results were also observed in the EDU, transwell, tube formation assays, and the protein expression levels of BAX, BCL-2, cyclinD1 and VEGFA; DAPT markedly suppressed the promotive effects on proliferation, migration, and angiogenesis of HUVECs induced by TRPM2-AS overexpression and Ts-exo (Figs. 5H, I and S5E, F).

### PABPC1 is the key molecule of TRPM2-AS in activating NOTCH1 signaling pathway

**Fig. 6:**
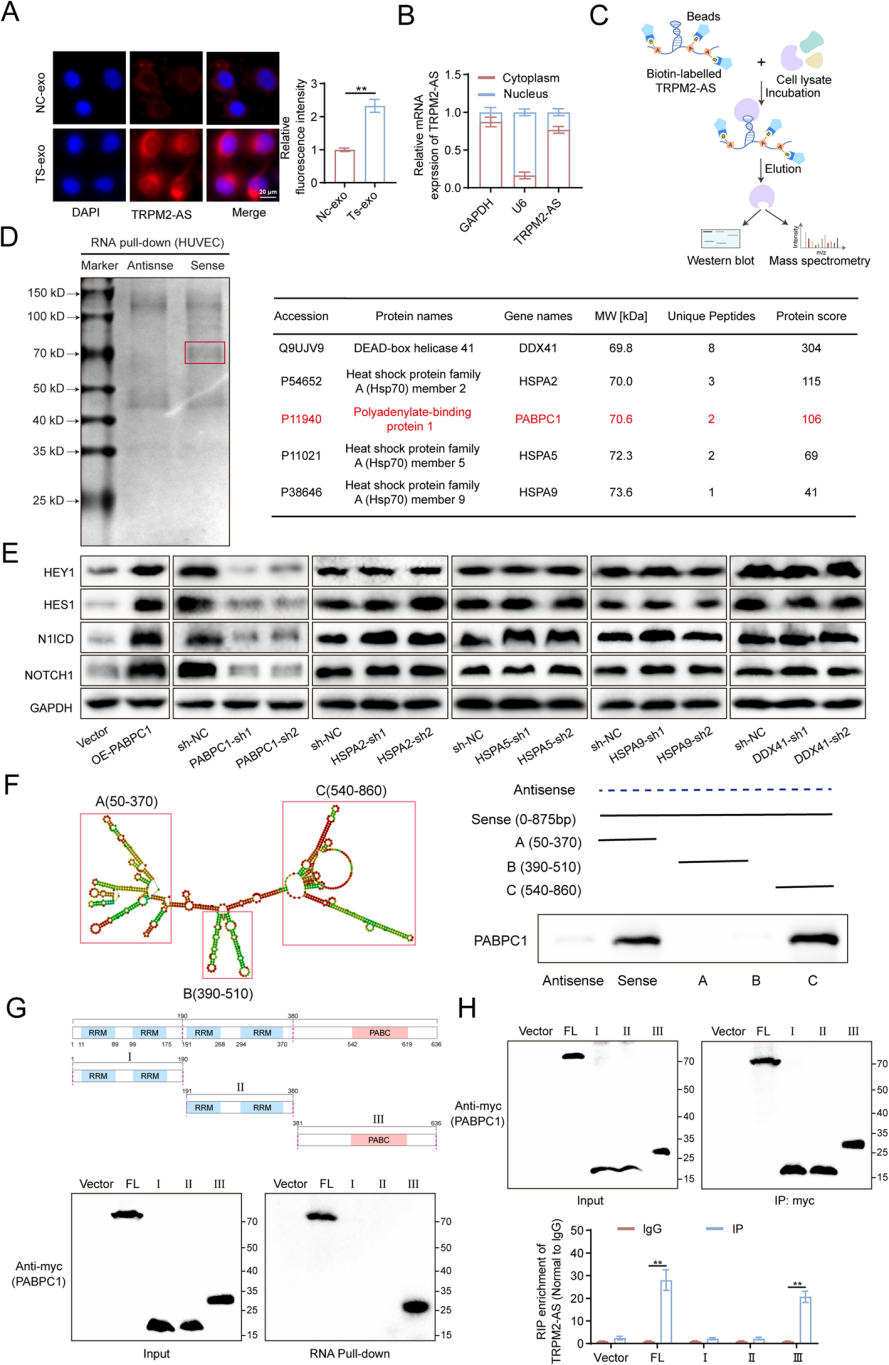
TRPM2-AS directly interacts with PABPC1. **A** FISH visualization revealing the subcellular localization of TRPM2-AS in HUVECs. Red fluorescence: Cy3-labeled TRPM2-AS; blue fluorescence: DAPI-stained nuclei. Scale bar: 20 μm. **B** RT-qPCR detecting the expression levels of TRPM2-AS in the cytoplasm and in nucleus respectively. **C**, **D** Schematic of RNA pull-down assay using biotin-labeled TRPM2-AS. The sense and antisense chains of biotin-labeled TRPM2-AS were synthesized as probes and combined with streptavidin-labeled magnetic beads. Proteins in HUVECs were extracted and incubated with beads coupled with RNA probe (**C**). RNA binding proteins were collected and stained with Coomassie bright blue. Mass spectrometry analysis confirming the proteins interacting with TRPM2-AS in HUVECs (**D**). **E** The expression level of NOTCH1 signaling pathway related proteins was identified by western blot with PABPC1/HSPA2/HSPA5/HSPA9/DDX41 knockdown. **F** The RNA secondary structure of TRPM2-AS was predicted using ViennaRNA Web Services (http://rna.tbi.univie.ac.at/) and RNA pull-down assays was conducted to confirm the domain of TRPM2-AS that interacted with PABPC1. **G**, **H** MYC-tag labeled full-length PABPC1 and its fragments I, II, III were overexpressed in HUVECs. RNA pull-down assay (**G**) and RIP assay (**H**) was used to confirm the exact fragment of PABPC1 that interacted with TRPM2-AS. Data were assessed with unpaired Student’s *t* test and presented as mean ± SD. ** *P* < 0.01

In line with GBC cells, FISH and nucleoplasm separation showed that in HUVECs, TRPM2-AS was mainly distributed in the cytoplasm (Fig. 6A, B). Because lncRNAs play a role by sponging miRNA (microRNA) or binding to proteins [21], we predicted 12 miRNAs that TRPM2-AS might sponge using lncbase (https://diana.e-ce.uth.gr/lncbasev3/), including miR-106b-5p, miR-17-5p, miR-210-3p, miR-22-3p, miR-27a-5p, miR-27b-3p, miR-30c-1-3p, miR-339-5p, miR-34a-5p, miR-4677-3p, miR-766-5p and miR-93-5p. After transfection of the mimic into HUVECs, western blot was used to detect the activation of the NOTCH1 signaling pathway. Unfortunately, none of these miRNAs appeared to have a meaningful effect on the NOTCH1 signaling pathway (Fig. S6A-C). Therefore, TRPM2-AS may activate the NOTCH1 signaling pathway by interacting with proteins. To test our hypothesis, we synthesized biotin-labeled TRPM2-AS full-length as a probe and conducted a RNA pull-down assay combined with mass spectrometry to identify the proteins that directly interacted with TRPM2-AS (Fig. 6C). Coomassie blue staining showed significant differences in protein expression levels at the 70 kD position compared to the antisense group. Interestingly, the molecular weights of DEAD-box RNA helicase 41 (DDX41), PABPC1, Heat shock protein A2 (HSPA2), Heat shock protein A5 (HSPA5), and Heat shock protein A9 (HSPA9) among the pull-down proteins were close to 70 kD (Fig. 6D). Therefore, we constructed cell lines with these five proteins stable knockdown and PABPC1 stable overexpression (Fig. S7A-C) and further detected activation of the NOTCH1 signaling pathway in HUVECs. Among these proteins, only PABPC1 knockdown inhibited the NOTCH1 signaling pathway, whereas PABPC1 overexpression activated the NOTCH1 signaling pathway (Fig. 6E). PABPC1 overexpression significantly promoted the proliferation, migration, and tube formation of HUVECs (Fig. S7D), whereas PABPC1 knockdown inhibited these effects (Fig. S7E). Furthermore, we overexpressed TRPM2-AS in HUVECs with PABPC1 knockdown. When PABPC1 was knocked down, the activation of the NOTCH1 signaling pathway (Fig. S8A, B) and the increase in proliferation, migration, and tube formation ability (Fig. S8C-E) of HUVECs by TRPM2-AS were no longer significantly different from those in the control group. These results demonstrate that PABPC1 acts as a crucial molecule in the activation of the NOTCH1 signaling pathway and in the promotion of proliferation, migration, and tube formation of HUVECs induced by TRPM2-AS.

These results encouraged us to study the specific domain of TRPM2-AS that interacted with PABPC1.Therefore, we used Vienna RNA Web Services (http://rna.tbi.univie.ac.at/) to predict the secondary structure of TRPM2-AS and then synthesized full-length biotin-labeled TRPM2-AS and its fragments A, B, and C as probes according to the secondary structure (Fig. 6F). Further, RNA pull-down assays and western blot analysis showed that PABPC1 could only be detected when full-length and fragment C were used as probes (Fig. 6F). This suggests that TRPM2-AS interacts with PABPC1 via domain C. In addition, to further identify the key domain of PABPC1 with which TRPM2-AS interacts, we constructed plasmids that overexpressed the myc-labeled full-length and fragments I, II, and III of PABPC1. RNA pull-down results showed that only full-length and fragment III of PABPC1 were pulled down by TRPM2-AS (Fig. 6G). We conducted RIP experiments using anti-Myc antibodies in HUVECs that overexpressing PABPC1 full-length and fragments I, II, and III. The RT-qPCR results indicated that TRPM2-AS was highly enriched in the PABPC1 full-length and fragment III groups (Fig. 6H). Taken together, these results suggest that TRPM2-AS interacts with fragment III of PABPC1 via domain C.

### TRPM2-AS enhances PABPC1-mediated inhibition of NUMB expression, thereby activating the NOTCH1 signaling pathway

**Fig. 7:**
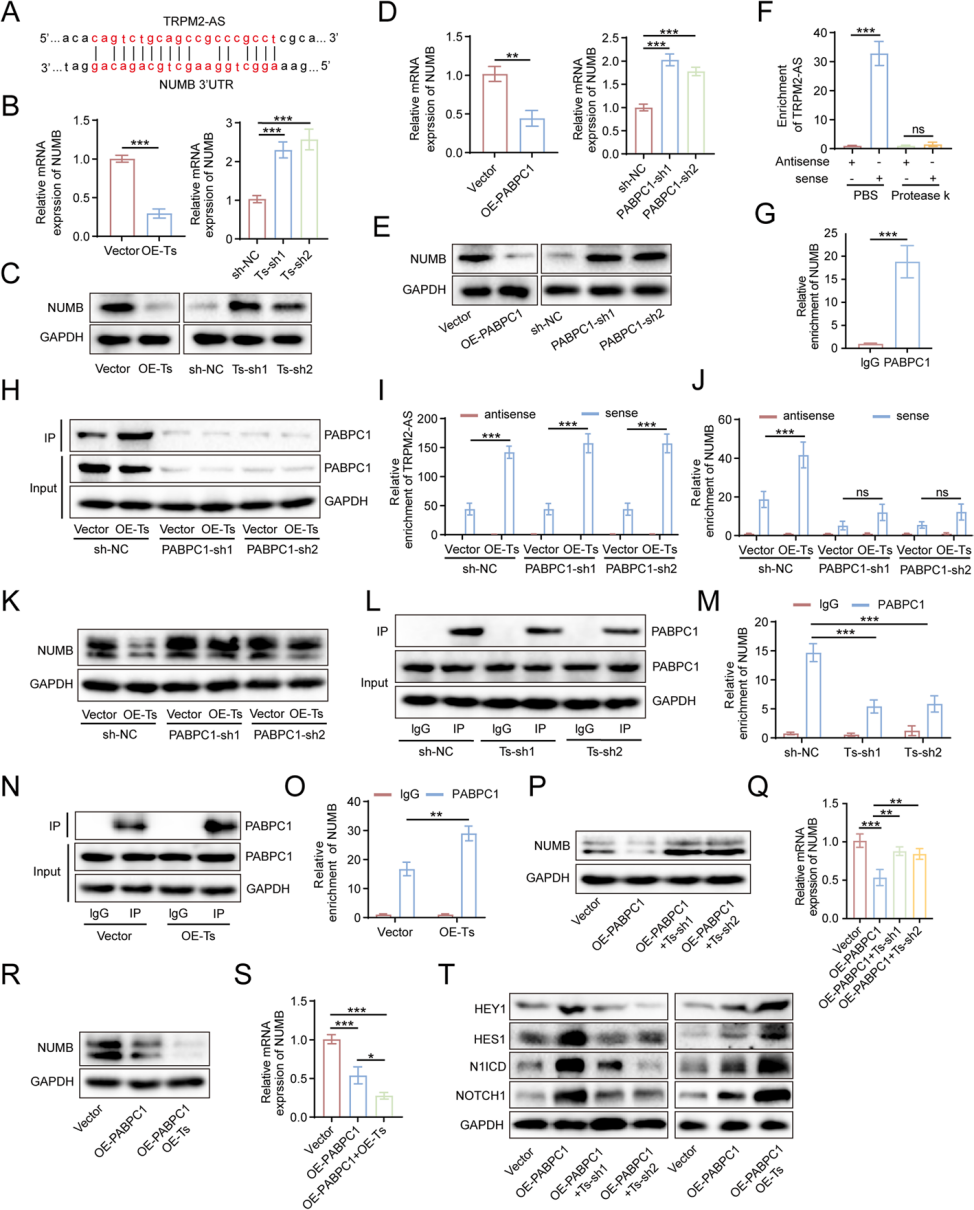
TRPM2-AS enhances PABPC1-mediated inhibition of NUMB expression. **A** The complementary pairing sequence of TRPM2-AS and NUMB mRNA 3'UTR. **B**, **C** RT-qPCR (**B**) and western blot (**C**) measuring the expression level of NUMB in the control group, TRPM2-AS overexpression group, and TRPM2-AS knockdown group. **D**, **E** The inhibitory effects of PABPC1 on NUMB were identified using RT-qPCR (**D**) and western blot (**E**). **F** RAP analysis of the relative enrichment of NUMB mRNA by biotin-labeled short DNA probe specifically complemented with TRPM2-AS with/without protease K treatment in substrate. **G** The direct interaction between PABPC1 and NUMB mRNA was confirmed using RIP analysis. **H**-**J** Negative control shRNA (sh-NC) and shRNA against PABPC1 (PABPC1-sh1/sh2) were transfected into HUVECs with/without TRPM2-AS overexpression. RAP followed by western blot/RT-qPCR analysis revealing the TRPM2-AS, PABPC1 protein and NUMB mRNA level pulled down by biotin-labeled short DNA probe specifically complemented with TRPM2-AS in different groups. **K** Western blot showing the inhibitory effects of TRPM2-AS on NUMB expression with/without PABPC1 knockdown. **L**-**O** RIP and RT-qPCR were used to quantify NUMB mRNA pulled down by PABPC1 in HUVECs with/without TRPM2-AS knockdown (**L**, **M**) and overexpression (**N**, **O**). **P**-**S** RT-qPCR and western blot analysis detecting the potentiating effect of TRPM2-AS on the inhibition of PABPC1 on NUMB. **T** Western blot showing the expression levels of NOTCH1 signaling pathway related proteins in control group cells and in PABPC1 knockdown group cells with/without TRPM2-AS overexpression/knockdown. Data were assessed with unpaired Student’s *t* test or one-way ANOVA and presented as mean ± SD. * *P* < 0.05; ** *P* < 0.01; *** *P* < 0.001; ns, no significance

As our results illustrated that PABPC1 is a critical molecule in TRPM2-AS activation of the NOTCH1 signaling pathway, we further explored how the TRPM2-AS-PABPC1 axis influences the NOTCH1 signaling pathway. We found that there was a complementary sequence between TRPM2-AS and the 3’UTR of the NUMB mRNA, a typical suppressor of the NOTCH1 signaling pathway (Fig. 7A). RT-qPCR and western blot showed that NUMB mRNA and protein expression levels decreased with the overexpression of TRPM2-AS and PABPC1, whereas the knockdown of TRPM2-AS and PABPC1 effectively increased NUMB mRNA and protein expression levels (Fig. 7B-E). We applied RAP assay to demonstrate the interaction between TRPM2-AS and NUMB mRNA. Treatment with protease K to remove the protein from the substrate significantly decreased the NUMB mRNA level pulled down by TRPM2-AS probe (Fig. 7F). Therefore, we confirmed that the interaction between TRPM2-AS and NUMB mRNA was dependent on protein rather than base complementary pairing. At the same time, we used RIP assay to detect the direct interaction between PABPC1 and NUMB mRNA, and the results proved that NUMB mRNA interacted directly with PABPC1 (Fig. 7G). Notably, PABPC1 served as a bridge between TRPM2-AS and NUMB mRNA.

We determined the probable impact of PABPC1 on the interaction between TRPM2-AS and NUMB mRNA. We overexpressed TRPM2-AS in control cells and cells with PABPC1 knockdown. Biotin-labeled TRPM2-AS probes were used to perform RAP experiments, and TRPM2-AS and NUMB mRNA and PABPC1 protein levels were detected in the pull-down products. The results showed that in the control group, TRPM2-AS overexpression significantly increased the levels of TRPM2-AS and NUMB in the pull-down products. However, when PABPC1 was knocked down, the level of TRPM2-AS in the pull-down products was still elevated in the TRPM2-AS overexpression group. In contrast, NUMB mRNA did not appear to differ from those of the control group (Fig. 7H-J). Our western blot results also verified that PAPBC1 is the intermediary molecule between TRPM2-AS with NUMB: The inhibitory effect of TRPM2-AS on NUMB protein expression disappeared with PABPC1 knockdown (Fig. 7K). Therefore, PABPC1 is indispensable for the interaction between TRPM2-AS and NUMB mRNA.

Although we confirmed that PABPC1 is the key molecule in TRPM2-AS-mediated inhibition of NUMB, whether TRPM2-AS affects the interaction between PABPC1 and NUMB mRNA remains unknown. RIP assay using anti-PABPC1 antibodies in HUVECs showed that the level of NUMB mRNA pulled down by PABPC1 significantly decreased with TRPM2-AS knockdown in HUVECs (Fig. 7L, M), while TRPM2-AS overexpression resulted in opposite results (Fig. 7N, O). Knockdown of TRPM2-AS further impaired PABPC1-mediated inhibition on NUMB expression (Fig. 7P, Q), NOTCH1 signaling pathway activation (Figs. 7T, S9A), and HUVECs angiogenesis (Fig. S9C), while overexpression of TRPM2-AS enhanced the inhibition of NUMB (Fig. 7R, S), activation of the NOTCH1 signaling pathway (Figs. 7T, S9B), and HUVECs angiogenesis (Fig. S9D) by PABPC1. In conclusion, TRPM2-AS enhanced the interaction between PABPC1 and the NUMB mRNA region, thereby strengthening the inhibition of PABPC1 on NUMB mRNA and ultimately activating the NOTCH1 signaling pathway to promote angiogenesis in GBC.

### PABPC1 inhibits NUMB expression through enhancing miRNA-mediated NUMB degradation

**Fig. 8:**
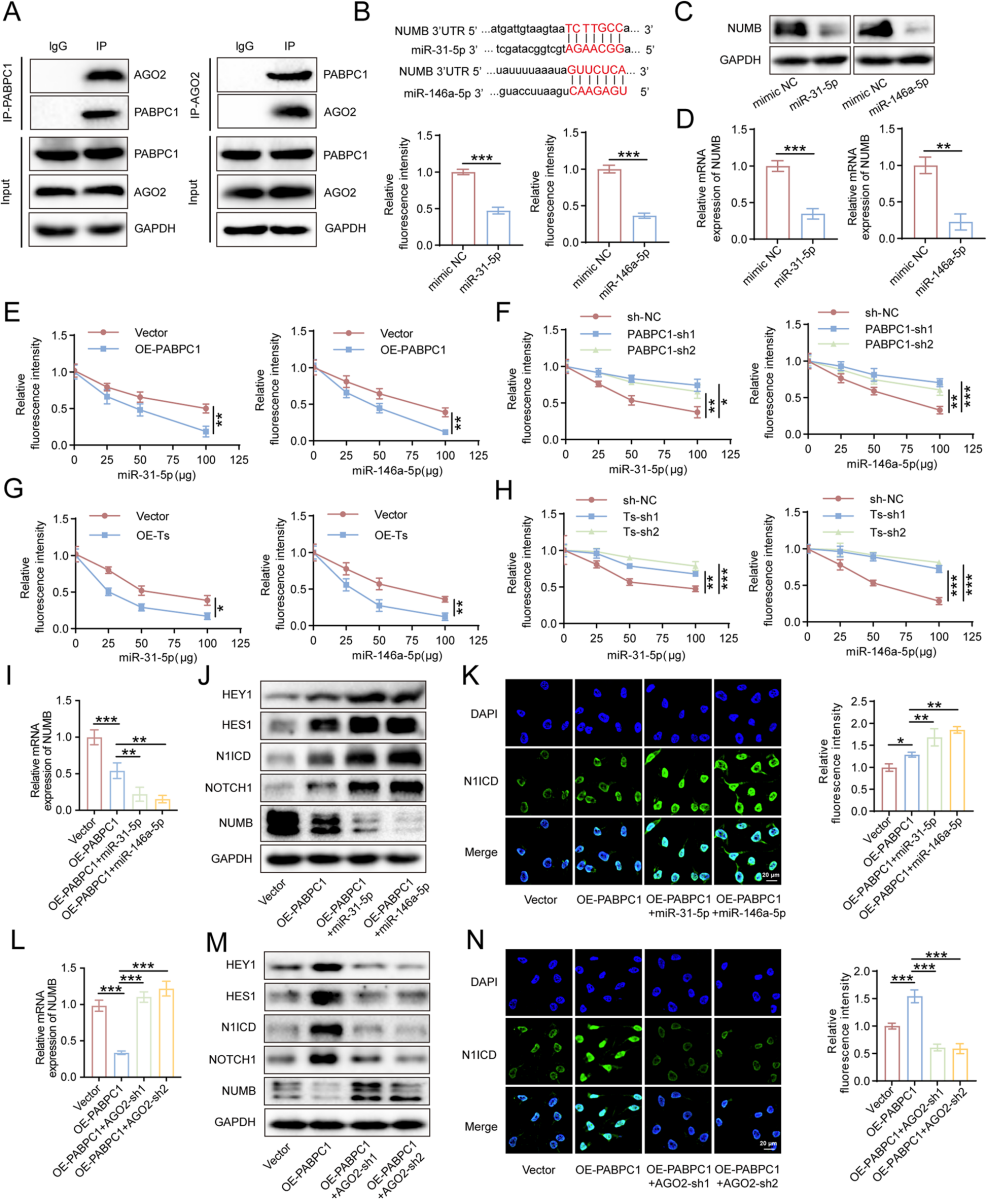
PABPC1 suppresses NUMB expression by enhancing miRNA-mediated NUMB degradation. **A** Direct interaction between PABPC1 and AGO2 in HUVECs was measured by Co-IP assay. **B** The site of NUMB 3’UTR bound by miR-31-5p and miR-146a-5p (upper). Dual-luciferase reporter system was performed to detect the interaction of miR-31-5p and miR-146a-5p with the NUMB 3’UTR (bottom). **C**, **D** Western blot (**C**), and RT-qPCR (**D**) confirming the degradation abilities of miR-31-5p and miR-146a-5p on NUMB. **E**–**H** Dual-luciferase reporter system showing the inhibition degree of NUMB by miR-31-5p and miR-146a-5p in HUVECs transfected with different amounts of miR-31-5p and miR-146a-5p with PABPC1 overexpression (**E**) or knockdown (**F**)/TRPM2-AS overexpression (**G**) or knockdown (**H**). **I**-**K** RT-qPCR (**I**), western blot (**J**), and immunofluorescence (**K**) measuring the activation of the NOTCH1 signaling pathway in the control group and in the PABPC1 overexpression group with/without miR-31-5p/miR-146a-5p treatment. Green fluorescence: corresponding primary antibody stained N1ICD; blue fluorescence: DAPI-stained nuclei. Scale bar: 20 μm. **L**-**N** RT-qPCR (**L**), western blot (**M**), and immunofluorescence (**N**) measuring the activation of the NOTCH1 signaling pathway in the control group and in the PABPC1 overexpression group with/without AGO2 knockdown. Data were assessed with unpaired Student’s *t* test or one-way ANOVA and presented as mean ± SD. * *P* < 0.05; ** *P* < 0.01; *** *P* < 0.001

PABPC1 plays an important role in NUMB mRNA inhibition. According to relevant reports, PABPC1 promotes mRNA translation by interacting with eIF3 and miRNA-mediated mRNA degradation through interaction with Argonaute risk component 2 (AGO2) [22]. As PABPC1 inhibited NUMB expression in HUVECs, we hypothesized that miRNA-mediated mRNA degradation might be involved in this process. Coimmunoprecipitation assays showed that PABPC1 directly interacted with AGO2 in HUVECs (Fig. 8A). Previous studies have shown that miR-31-5p and miR-146a-5p target NUMB mRNA and thus mediate NUMB degradation [23, 24]. In HUVECs, dual-luciferase reporter assay results indicated that miR-31-5p and miR-146a-5p bound to NUMB mRNA (Fig. 8B). RT-qPCR and western blot assays demonstrated that both the mRNA and protein levels of NUMB were downregulated by transfection with miR-31-5p and miR-146a-5p mimics (Fig. 8C, D). To confirm that the efficiency of miRNA-mediated NUMB mRNA degradation depends on PABPC1, we gradually increased the transfection dose of miR-31-5p and miR-146a-5p mimics in HUVECs with PABPC1 overexpression or knockdown and then plotted the inhibition curve of the miRNA on NUMB mRNA. PABPC1 overexpression significantly promoted the inhibitory effects of miR-31-5p and miR-146a-5p (Fig. 8E), which were diminished by PABPC1 knockdown (Fig. 8F). Similar results were observed in cell lines overexpressing or with TRPM2-AS knockdown. After overexpression of TRPM2-AS, the inhibitory effects of miR-31-5p and miR-146a-5p increased (Fig. 8G), whereas TRPM2-AS knockdown resulted in the opposite effects (Fig. 8H). In addition, we increased the expression of miR-31-5p and miR-146a-5p based on PABPC1 overexpression to verify whether miR-31-5p and miR-146a-5p affected the inhibition of NUMB expression, activation of the NOTCH1 signaling pathway, and the promoting effect of PABPC1 on HUVECs angiogenesis. As expected, miR-31-5p and miR-146a-5p improved these functions of PABPC1 (Figs. 8I-K, S10A). Moreover, when AGO2 was knocked down to restrict miRNA-mediated mRNA degradation, PABPC1-mediated inhibition of NUMB expression, NOTCH1 signaling pathway activation, and HUVECs angiogenesis were significantly restricted (Figs. 8L-N, S10B). These results demonstrated that PABPC1 inhibits NUMB expression through miRNA-mediated NUMB degradation.

### Loss of TRPM2-AS improves the inhibitive effects of NOTCH1 inhibitor DAPT on GBC angiogenesis in vivo

**Fig. 9:**
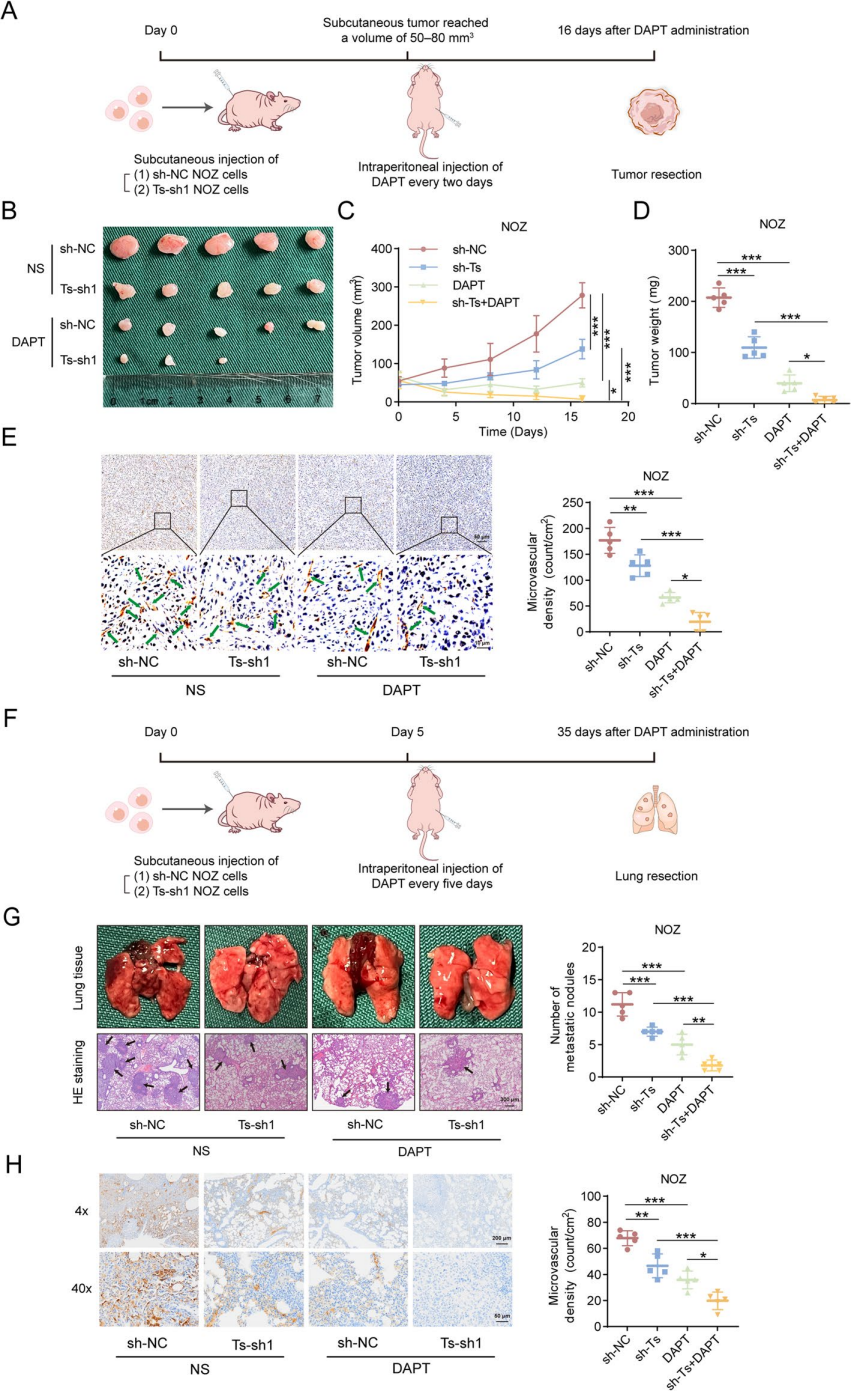
TRPM2-AS knockdown enhances the suppressive effects of DAPT on GBC tumors. **A** 2 × 10^6^ NOZ cells with/without TRPM2-AS knockdown were injected subcutaneously into nude mice. When tumor volume increased to 50–80 mm^3^, normal saline (NS) or DAPT (20 mg/kg) were injected intraperitoneally once every 2 days. Then the nude mice were euthanized, and the tumors were resected after 16 days. **B**-**D** Representative images and quantification of surgically removed GBC tumors in the control group and in the TRPM2-AS knockdown group with/without DAPT treatment (*n* = 5). **E** Immunohistochemical staining of microvascular density in disserted GBC tumors. Scale bar: 10/50 μm. **F** The lung metastasis model was constructed by injecting of 2 × 10^6^ control or TRPM2-AS knockdown NOZ cells intravenously, and the nude mice were euthanized, and the lung tissues were resected after 40 days by injection of saline or DAPT (20 mg/kg) once every 5 days. **G** Representative images of surgically removed lung tissues and H&E staining revealing lung metastasis in the control group and in the TRPM2-AS knockdown group with/without DAPT treatment. Scale bar: 300 μm (*n* = 5). **H** Immunohistochemical staining for detection of the microvascular density in disserted lung tissues. Scale bar: 50/200 μm. Data were assessed with one-way ANOVA or two-way ANOVA and presented as mean ± SD. * *P* < 0.05; ** *P* < 0.01; *** *P* < 0.001

Having demonstrated that TRPM2-AS promoted GBC angiogenesis by activating NOTCH1 signaling pathway, we hypothesized that TRPM2-AS knockdown might have a synergistic effect on the inhibitory effect of DAPT on GBC angiogenesis. To this end, we injected BALB/c nude mice with control GBC cells and TRPM2-AS knockdown GBC cells, and selectively used DAPT in mouse xenograft and lung metastasis models (Fig. 9A, F). As TRPM2-AS knockdown or DAPT alone limited tumor growth and decreased microvascular density in resected tumors, the combined treatment of the two resulted in a more pronounced reduction in the endpoints of tumor weight and volume (Fig. 9B-D), as well as microvascular density (Fig. 9E). As for the lung metastasis of GBC cells in nude mice, compared to the TRPM2-AS alone knockdown group and the DAPT-alone treatment group, H&E staining assays indicated that the combined treatment of the two reduced metastatic nodules in the lung tissue (Fig. 9G). Furthermore, immunohistochemical staining showed the lowest microvascular density in the TRPM2-AS and DAPT combined treatment group compared to the control, TRPM2-AS knockdown, and DAPT groups (Fig. 9H). TRPM2-AS knockdown considerably improved the inhibitory effects of the NOTCH1 blocker DAPT on GBC angiogenesis in xenograft and lung metastasis models.

## Discussions

We are fully aware of the enormous difficulties facing the current clinical treatment of GBC and are committed to finding a new target and elucidating its molecular mechanisms in detail. In recent years, with advancements in next-generation sequencing, novel systemic therapies, and immunotherapies, the treatment paradigm for cancer and prognostic statistics have gradually changed, bringing great opportunities for the treatment of GBC. Increasing evidence has shown that exosomal lncRNAs transfer pro-angiogenic signals from cancer cells to endothelial cells to remodel the tumor microenvironment and promote angiogenetic activity in several types of cancer [25]. Furthermore, exosomal lncRNAs can act as diagnostic and prognostic biomarkers for cancer [26]. However, to the best of our knowledge, no single study has identified the role of exosomal lncRNAs in promoting tumor angiogenesis in GBC or the precise molecular mechanism underlying neovascularization. In our study, we identified that the lncRNA TRPM2-AS is overexpressed in GBC tissues and cell lines through post-transcriptional m^6^A modifications and is associated with the prognosis of GBC patients. We then identified that GBC cells secrete the exosomal lncRNA TRPM2-AS, which communicates with endothelial cells to promote angiogenesis via activation of the NOTCH1 signaling pathway. Furthermore, we found that PABPC1 was involved in miRNA-mediated NUMB mRNA degradation via interaction with AGO2, and that TRPM2-AS enhanced the interaction between PABPC1 and NUMB mRNA, ultimately leading to activation of the NOTCH1 signaling pathway.

TRPM2-AS, the antisense lncRNA identified in our study, is involved in cancer progression, acts as a therapeutic or prognostic biomarker, and is involved in drug resistance [27]. Exosomes are a type of cancer-related signal transduction, and their extraction can easily detect the underlying activity of the tumor microenvironment and has great potential as diagnostic and prognostic biomarkers [26]. Moreover, exosomes have a longer half-life and can be easily extracted from body fluids such as blood, urine, milk, and cerebrospinal fluid, indicating the feasibility of using exosomal biomarkers in clinical practice [28]. Because early diagnosis is crucial for improving the resectability and survival outcomes of GBC, the feasibility of using exosomes as diagnostic biomarkers will certainly improve the early diagnosis rate and survival outcomes. Some studies have shown that exosomal lncRNAs are involved in carcinogenesis and are potential diagnostic and prognostic biomarkers for GBC [29]. However, none of these studies have explored the role of exosomal lncRNAs in inducing angiogenesis in GBC. Therefore, we expect, with further validation, exosomal TRPM2-AS can be used as a diagnostic or prognostic biomarker for GBC.

Since our data confirmed the potential clinical value and therapeutic prospects of TRPM2-AS in GBC, understanding the precise molecular mechanism of TRPM2-AS upregulation in GBC is crucial for exploring target points for novel treatment strategies. We found that post-transcriptional m^6^A modifications led to the overexpression of TRPM2-AS in GBC. Post-transcriptional m^6^A modifications are involved in the pathogenesis of human cancers and their regulatory and physiological functions [30]. Further experiments revealed that the binding of IGF2BP2 to the methylated 271 site of TRPM2-AS resulted in the overexpression of TRPM2-AS. This interplay between methyltransferases and binding proteins is crucial for maintaining post-transcriptional m^6^A modification and leads to the continuous overexpression and stability of TRPM2-AS in GBC. Moreover, both TRPM2-AS and IGF2BP2 were associated with patient survival in our study, suggesting a synergistic effect on GBC pathogenesis and, therefore, prognosis. In addition, two previously published studies explored the role of post-transcriptional m^6^A modification in RNA and its associated methyltransferases in the carcinogenesis of GBC [31, 32].

Angiogenesis is a crucial physiological process for the growth, invasion, and metastasis of tumors, and abnormal angiogenic activity is associated with chemoresistance [33]. Therefore, drugs that target or block angiogenesis to inhibit tumor growth and invasion are considered a new frontier in cancer treatment [33]. Clinical evidence suggests that targeting the pathways involved in promoting angiogenesis can be used for the treatment of cancers [34]. Treatment of HUVECs with TRPM2-AS overexpressed exosomes not only increased the cell proliferation, migration, and tube formation, but the subsequent xenograft and lung metastasis experiment also showed that treatment with TRPM2-AS overexpressed exosomes aggravated the tumor angiogenesis, thereby promoting further tumor growth and metastasis. Angiogenesis is a decisive prerequisite for tumor malignancy and increased microvascular density (MVD) is associated with an aggressive tumor phenotype and poor prognosis. Targeting angiogenesis is a promising avenue for tumor treatment, and there are indeed several drugs still under investigation [35]. Therefore, comprehensive genomic profiling with appropriate targeted therapies directed against neovascularization may improve antitumor activity and survival outcomes.

Although the involvement of the NOTCH1 pathway in several solid tumors has been investigated, its involvement in the regulation of pathological angiogenesis in GBC has not been fully elucidated. However, accumulating evidence supports the involvement of the NOTCH1 pathway in biliary tract carcinogenesis [36]. Studies on GBC are limited, with some reporting the involvement of NOTCH (1-4) signaling or its downstream factors in carcinogenesis, and the association of NOTCH1 family proteins and their endothelial ligands with poor prognosis [37, 38]. However, none of these studies have investigated the effect of NOTCH signaling in inducing GBC angiogenesis. It is well known that protein-RNA interactions determine the fate of various cellular processes and are linked to several diseases, including carcinogenesis [39]. Just as acting as a microRNA sponge is critical for many lncRNAs to exert regulatory functions in tumor progression, TRPM2-AS has also been shown to sponge microRNA-138-5p to activate epidermal growth factor receptor (EGFR) and phosphatidylinositol 3-kinase (PI3K)/AKT signaling to promote the malignant biological behavior of non-small cell lung cancer (NSCLC) cells [40]. Intriguingly, we found that the promoting effects of TRPM2-AS on the NOTCH1 signaling pathway were independent of the miRNA sponge mechanism. In our study, TRPM2-AS mediated binding of PABPC1 to the poly(A) tail of NUMB mRNA resulted in the loss of NUMB-mediated inhibition of NOTCH1 signaling. NUMB, an inhibitor of NOTCH signaling, blocks the NOTCH transmembrane receptor and is downregulated in several tumors [41]. PABPC1 consists of four RNA binding domains which can recognize 3’poly (A) tail and eIF4F complex on mRNA 5′ cap to mediate circularization of mRNA, a linker region which promotes PABPC1-mRNA association, and a C-terminal MLEE domain which recognized and binds to a peptide motif called PABP-interacting Motif 2 (PAM2) to involve in mRNA processing and translation [42]. Multiple PAM2 motif-containing proteins (PACs) compete with each other to bind to the MLEE domain of PABPC1, these PCPs determines mRNA fate [42]. Among several PCPs, GW182 interacts with AGO to form an miRNA-induced silencing complex to mediate mRNA silencing either by repressing translation or promoting the deadenylation of mRNA [43]. This AGO-GW182 silencing complex has a strong affinity for the C-terminal MLEE domain of PABPC1, which ultimately results in silencing of the target mRNA [44]. Based on the results of our study and the evidence from previous reports, the RNA binding domain of PABPC1 recognizes and binds on the 3’poly (A) tail and eIF4F complex on 5′ cap of NUMB and the C-terminal MLEE domain of PABPC1 recruited AGO-GW182 miRISC which ultimately resulted in silencing of NUMB. MiRNA-mediated mRNA degradation is a typical way to regulate protein expression levels, and previous studies have identified that miR-31-5p and miR-146a-5p downregulate NUMB expression [23, 24]. miR-31-5p is highly expressed and functions as an oncogenic miRNA in colorectal cancer (CRC), and the negative regulation of miR-31-5p on NUMB results in increased proliferation, invasion, and migration of CRC cells [23]. In addition, miR-146a-5p targets the 3’UTR of NUMB, which is highly similar across various species [24]. Our results reveal an inhibitory effect of PABPC1 and AGO2 on NUMB that requires miR-31-5p and miR-146a-5p mediated degradation. We suppose that miR-31-5p and miR-146a-5p are two carcinogenic miRNAs in GBC and might directly influent the malignant biological behaviors of GBC cells, but more convincing data are needed to further support this viewpoint. In summary, the expression of TRPM2-AS and PABPC1 can indirectly result in the activation of NOTCH1 signaling through miRNA-mediated NUMB mRNA degradation [41, 45].

We identified a prominent role of the NOTCH1 pathway in regulating tumor angiogenesis. Targeting molecules of the NOTCH1 pathway may provide insight into exploring novel drug targets for tumor angiogenesis. Recently, multiple drugs targeting NOTCH signaling, its precursors, and downstream factors have been tested in preclinical studies and clinical trials. The most frequently used drug γ-secretase inhibitors (GSIs) have shown anticancer effects against hepatocellular cancer, lung cancer, colorectal cancer, breast cancer, prostate cancer, acute myeloid leukemia (AML), or gliomas in preclinical studies as well as early phase trials [46]. Interestingly, some γ-secretase modulators have specifically shown anti-NOTCH1 activity by causing cell cycle arrest in AML with reduced adverse events [47]. Brontictuzumab, an antibody that directly targets NOTCH1, has some clinical benefits in the treatment of lymphoid malignancies and refractory solid tumors [48]. We may obtain more surprising results with a combination therapy targeting multiple signaling pathways that block the crosstalk between NOTCH1 and other signaling pathways, such as EGFR or VEGF. We found that γ-secretase inhibitor DAPT restricted tumor angiogenesis in GBC, which is consistent with previous research results [49]. More importantly, combined deletion of TRPM2-AS enhanced the inhibitory effects of DAPT. This encouraging result suggests that the use of DAPT or other NOTCH1 inhibitors in GBC patients with low TRPM2-AS expression can achieve the desired antiangiogenic effect. Taken together, we conclude that targeting NOTCH1 signaling could be an attractive therapeutic strategy against cancer over the next decade. With new next-generation single-cell RNA sequencing techniques, we can obtain further accurate information regarding tumor angiogenesis through the transcriptomes of thousands of GBC cells. In addition, there are emerging concepts regarding the role of tumor angiogenesis and the theory of vessel co-option, resulting in alternative vascularization of tumors to induce chemoresistance, which should be further explored [50].

Our study has several limitations. First, we did not perform in vivo experiments to test whether PABPC1 recruits AGO2 to form a miRNA-silencing complex that inhibits NUMB mRNA. Second, we did not use a patient-derived xenograft model to validate angiogenesis. Third, our study did not focus on if the TRPM2-AS-related axis identified in our study is related to vessel co-option in GBC. Fourth, we did not explore the role of TRPM2-AS in chemoresistance induction. Finally, the prognosis of patients with GBC based on TRPM2-AS expression requires further validation using multicenter data. Despite these limitations, we believe that the molecular mechanisms and pathways identified in our study will certainly help understand the carcinogenesis of GBC and explore novel treatment strategies.

## Conclusions

**Fig. 10:**
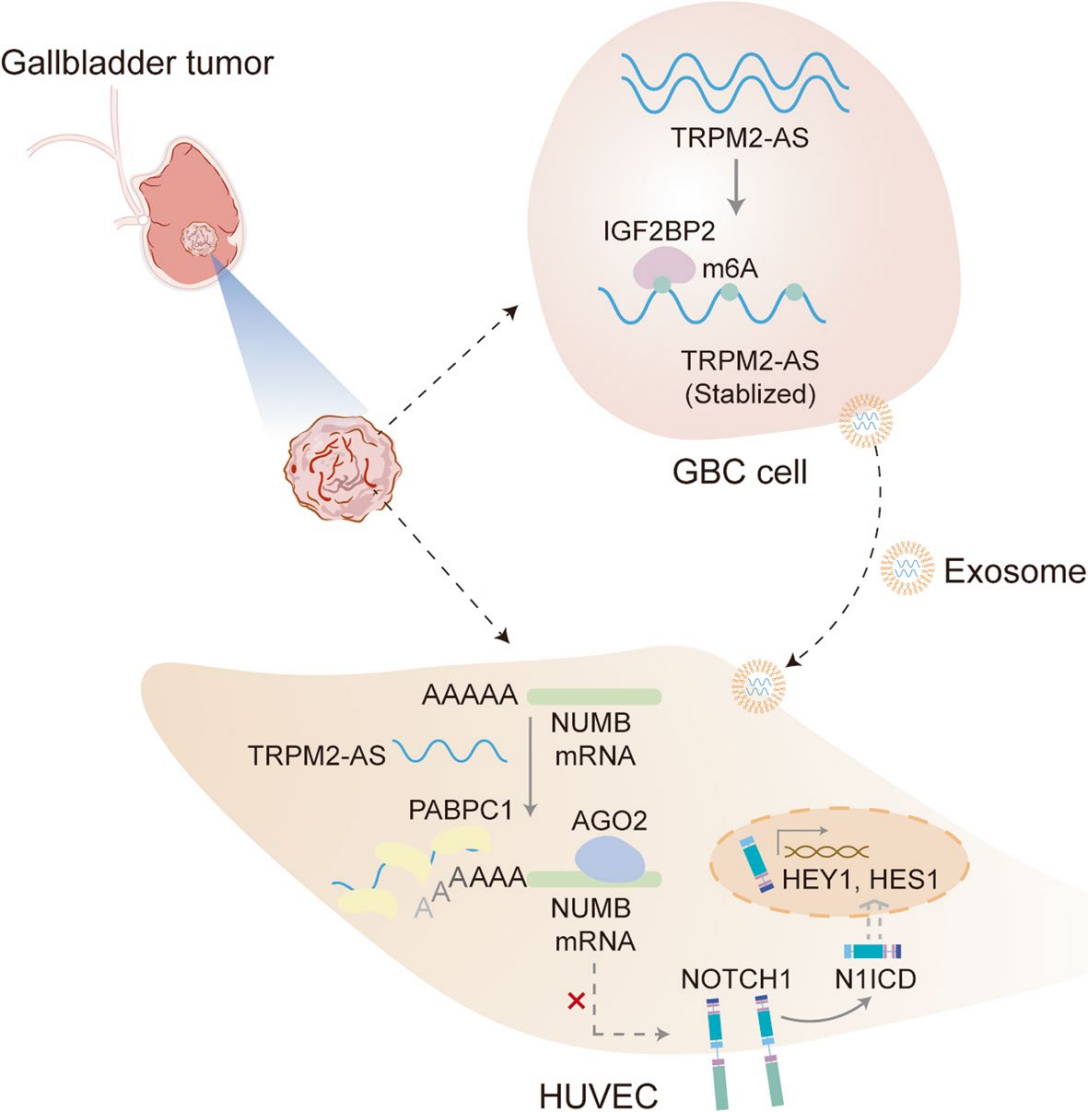
Schematic of the role of the IGF2BP2-TRPM2-AS/PABPC1-NUMB-NOTCH1 axis in GBC angiogenesis. In GBC cells, IGF2BP2 increases the stability of TRPM2-AS in an m^6^A-dependent manner, and overexpressed TRPM2-AS in tumors can be transferred to endothelial cells via exosomes and promote endothelial cells angiogenesis by activating the NOTCH1 signaling pathway. Mechanistically, TRPM2-AS promotes the degradation of NUMB mRNA by enhancing the interaction of PABPC1 with NUMB mRNA. Further studies have shown that PABPC1 interacts with AGO2 and then inhibits the expression of NUMB by promoting miR-31-5p and miR-146a-5p-mediated NUMB degradation

In conclusion, our data elucidate the critical role of TRPM2-AS in GBC angiogenesis and demonstrate that IGF2BP2-TRPM2-AS/ PABPC1-NUMB-NOTCH1 is a novel pro-angiogenic axis in GBC. TRPM2-AS overexpression positively correlated with angiogenesis and poor prognosis in patients with GBC. IGF2BP2 ensures the stability and high expression of TRPM2-AS via an m^6^A dependent mechanism. Notably, TRPM2-AS is transported from GBC cells to HUVECs, where it strengthens the inhibitory effect of PABPC1 on the expression of NUMB mRNA, promotes the activation of NOTCH1 signaling pathway, and ultimately induces the proliferation and migration of HUVECs. PABPC1 inhibits NUMB mRNA expression through interacting with AGO2 and promotes miR-31-5p and miR-146a-5p-mediated degradation of NUMB mRNA (Fig. 10). Targeting TRPM2-AS is expected to be a promising strategy to suppress GBC tumor angiogenesis and may provide broader applications for future GBC therapy.

### Supplementary Information

**Supplementary Material 1. **

## Data Availability

Data is provided within the manuscript or supplementary information files.

## Abbreviations

GBC: Gallbladder cancer
TNBC: Triple-negative breast cancer
NSCLC: Non-small cell lung cancer
AML: Acute myeloid leukemia
CRC: Colorectal cancer
LncRNA: Long non-coding RNA
miRNA: MicroRNA
shRNA: Short hairpin RNA
TRPM2-AS: Transient receptor potential cation channel subfamily M member 2 antisense RNA
HUVECs: Human umbilical vein endothelial cells
HEGICs: Human gallbladder epithelial immortalized cells
m^6^A: N6-methyladenosine
IGF2BP1: Insulin like growth factor 2mRNA binding protein 1
IGF2BP2: Insulin like growth factor 2mRNA binding protein 2
IGF2BP3: Insulin like growth factor 2mRNA binding protein 3
YTHDF1: YTH N6-methyladenosine RNA binding protein 1
YTHDF3: YTH N6-methyladenosine RNA binding protein 3
N1ICD: NOTCH1 intracellular domain
PABPC1: Poly(A) binding protein cytoplasmic 1
EGFR: Epidermal growth factor receptor
VEGF: Vascular endothelial growth factor
VEGFR: Vascular endothelial growth factor receptor
VEGFA: Vascular endothelial growth factor A
ICAM1: Intercellular adhesion molecule 1
FoxM1: Fork head box M1
TAF15: TATA-box-binding protein-associated factor 15
DDX41: DEAD-box RNA helicase 41
HSPA2: Heat shock protein A2
HSPA5: Heat shock protein A5
HSPA9: Heat shock protein A9
AGO2: Argonaute risk component 2
NF-κB: Nuclear factor-κB
BCL-2: B-cell chronic lymphocytic leukemia/lymphoma-2
BAX: BCL-2-associated X protein
EGFR: Epidermal growth factor receptor
PI3K: Phosphatidylinositol 3-kinase
PACs: PAM2 motif-containing proteins
3’UTR: 3’ Untranslated region
IHC: Immunohistochemistry
H&E: Hematoxylin and eosin
IF: Immunofluorescence
RT-qPCR: Reverse transcription-quantitative polymerase chain reaction
Co-IP: Co-immunoprecipitation
RIP: RNA immunoprecipitation
RAP: RNA antisense purification
MeRIP: Methylated RNA immunoprecipitation sequencing
FISH: Fluorescence in situ hybridization
ChIP: Chromatic immunoprecipitation
TNM: Tumor node metastasis
MVD: Microvascular density
CA19-9: Cancer antigen 19–9
OS: Overall survival
RFS: Recurrence-free survival
DMEM: Dulbecco's modified Eagle’s medium
RPMI: Rosewell Park Memorial Institute
FBS: Fetal bovine serum
EDU: 5-Ethynyl-2’-deoxyuridine
DAA: Deazaadenosine
DAPT: N-[N-(3, 5-difluorophenyl)-l-alanyl] -s-phenylglycine t-butyl ester
KEGG: Kyoto Encyclopedia of Genes and Genomes
