# Supplementary material for: Exosomal long non-coding RNA TRPM2-AS promotes angiogenesis in gallbladder cancer through interacting with PABPC1 to activate NOTCH1 signaling pathway
Source: Mol Cancer. 2024 Mar 27;23:65. doi: 10.1186/s12943-024-01979-z (PMC10967197; doi:10.1186/s12943-024-01979-z)
Supplement: Supplementary file 1 — Supplementary Material 1. [file 12943_2024_1979_MOESM1_ESM.docx]

**Supplementary Figures**


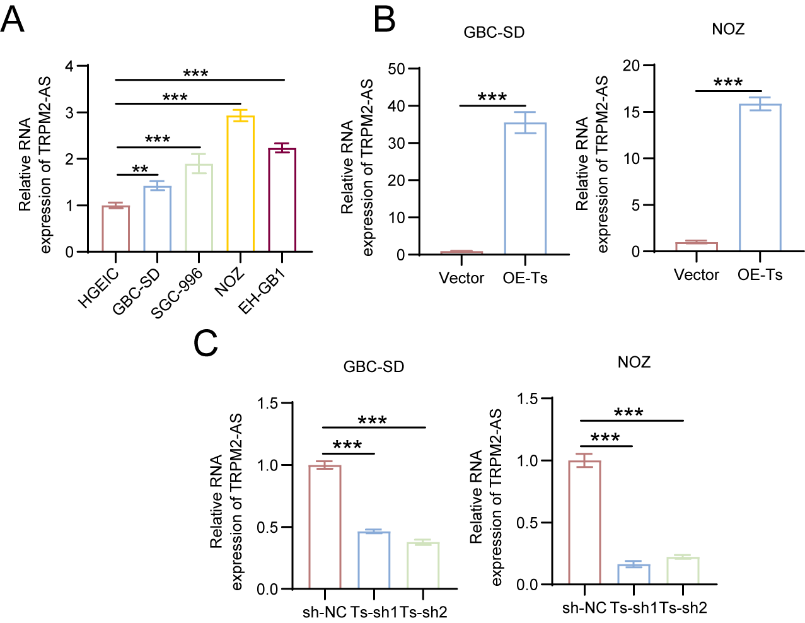


**Figure S1 Construction of TRPM2-AS overexpression/knockdown GBC-SD and NOZ cells.**

**(A)** RT- qPCR assessment of TRPM2-AS expression level in HGEICs and GBC cells (GBC-SD/SGC-996/NOZ/EH-GB1). **(B-C)** RT-qPCR assessment of TRPM2-AS expression levels in GBC-SD and NOZ cells with TRPM2-AS overexpression **(B)** and knockdown **(C)** using lentivirus transfection.

Data were assessed with unpaired Student’s *t* test or one-way ANOVA and presented as mean ± SD. ** *P*< 0.01; *** *P* < 0.001.

**
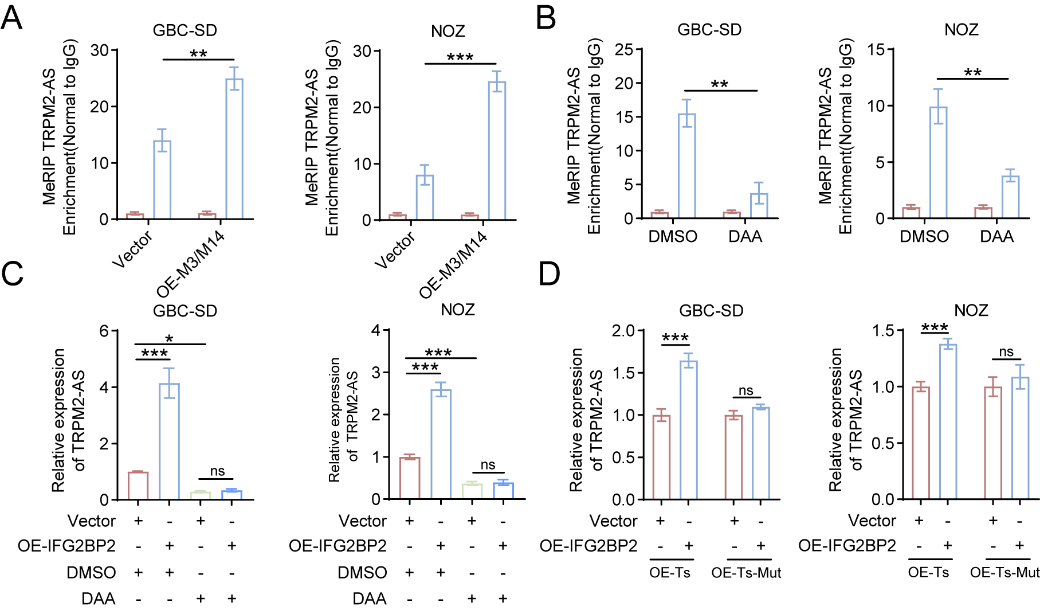
**

**Figure S2 IFG2BP2 regulates the stability and expression of TRPM2-AS in an m^6^A-dependent manner.**

**(A-B)** MeRIP assays showing the enrichment of TRPM2-AS in GBC-SD and NOZ cells with/without Mettle3/ Mettle14 (M3/M14) overexpression **(A)** and DAA treatment **(B)**. **(C)** RT-qPCR quantification of the relative expression of TRPM2-AS with/without the treatment of IGF2BP2 overexpression and DAA. (**D**) IGF2BP2 was overexpressed in GBC cells with wild or mutant TRPM2-AS overexpression, and the RNA levels of TRPM2-AS were detected by RT-qPCR.

Data were assessed with unpaired Student’s *t* test or one-way ANOVA and presented as mean ± SD. * *P* < 0.05; ** *P*< 0.01; *** *P* < 0.001; ns, no significance.

**
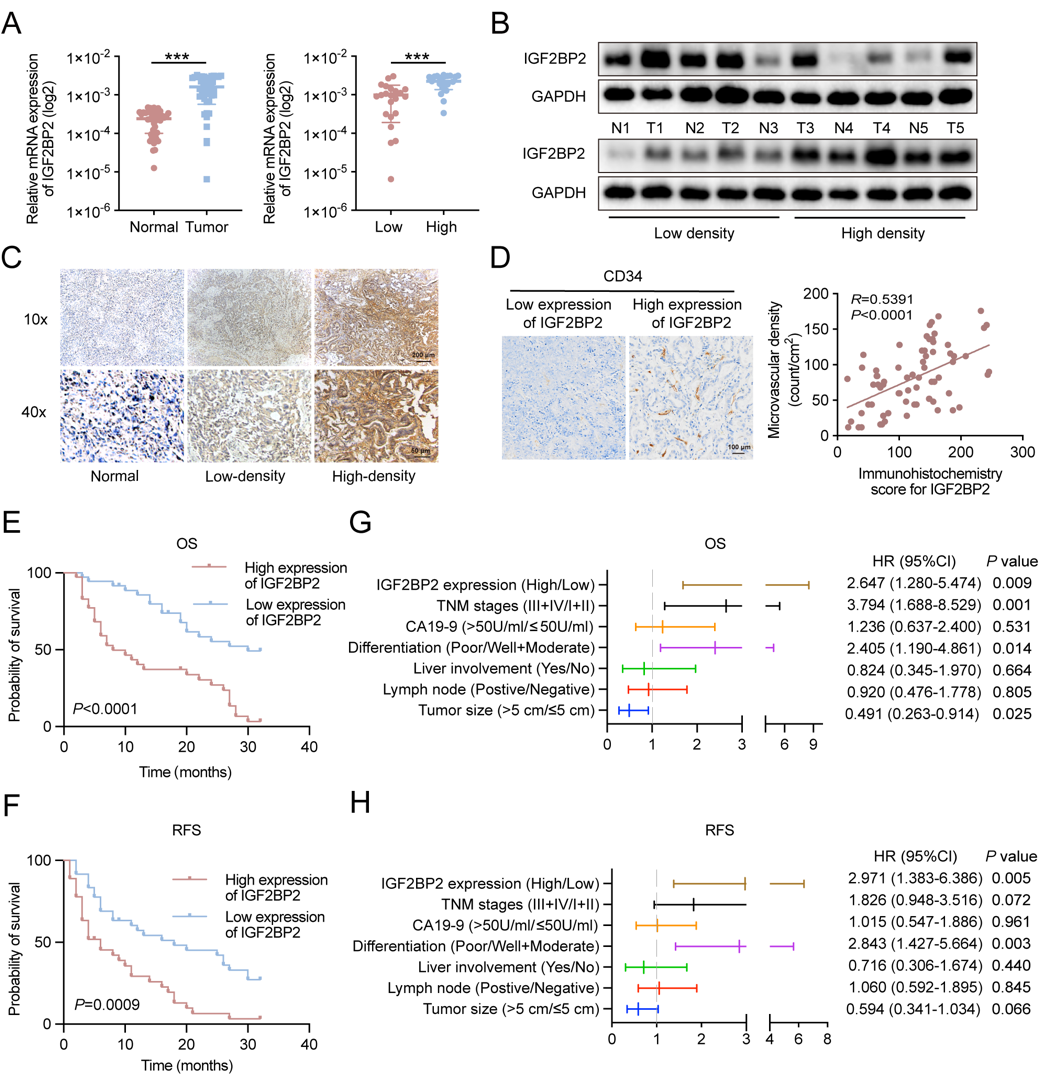
**

**Figure S3 IGF2BP2 is overexpressed in GBC patients and associated with poor prognosis.**

**(A-C)** Expression levels of IGF2BP2 in normal tissues, GBC tissues with low microvascular density and GBC tissues with high microvascular density from GBC patients were determined by RT-qPCR **(A)** western blot **(B)** and immunohistochemistry **(C)**. Scale bar: 50/200 μm. **(D)** Representative images of the immunohistochemical staining of CD34 in tissues with high/low IGF2BP2 expression and Pearson’s correlation analysis of the positive correlation between IGF2BP2 expression and microvascular density. Scale bar: 100 μm. **(E-F)** Kaplan–Meier curve analysis of the correlation between IGF2BP2 expression level and overall survival (OS)/recurrence-free survival (RFS) of GBC patients. **(G-H)** Multivariate analysis of the prognostic factors for OS and RFS of GBC patients.

Data were assessed with unpaired Student’s *t* test and presented as mean ± SD. *** *P* < 0.001.


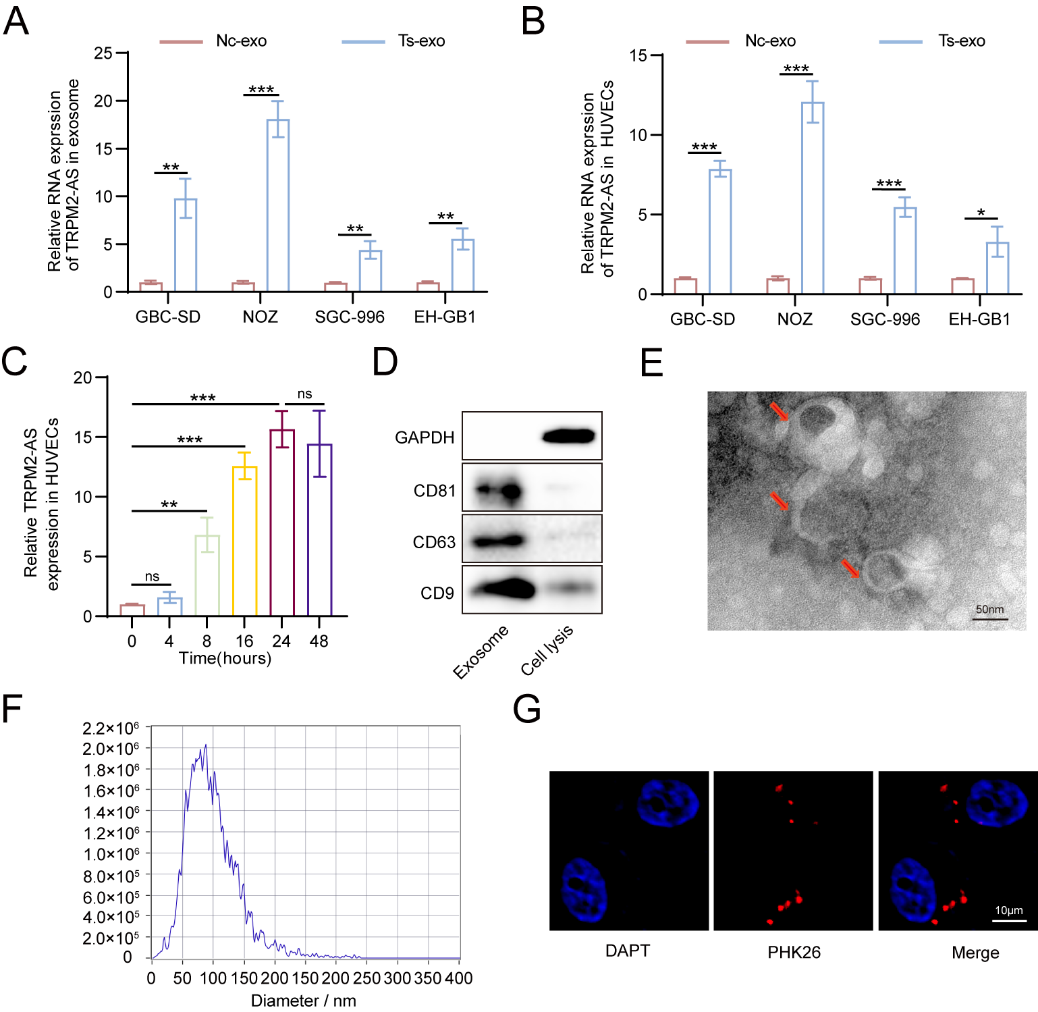


**Figure S4 Exosomes transport TRPM2-AS to HUVECs to promote angiogenesis.**

**(A)** RT-qPCR showing the relative expression level of TRPM2-AS in exosomes from GBC-SD/NOZ /SGC-996/EH-GB1 cells with/without TRPM2-AS overexpression. **(B)** RT-qPCR showing the relative expression level of TRPM2-AS in HUVECs cultured in Nc-exo and Ts-exo from GBC-SD/NOZ/SGC-996/EH-GB1 cells. Nc-exo: exosomes from negative control GBC cells. Ts-exo: exosomes from TRPM2-AS overexpressing GBC cells. **(C)** RT-qPCR assessment of TRPM2-AS level in HUVECs cultured with TRPM2-AS-containing exosomes for 0, 4, 8, 16, 24, and 48 hours. **(D)** Western blot validation of GAPDH, CD81, CD63, and CD9 in exosomes and NOZ cells. **(E)** Representative images of exosome structure captured by electron microscopy. Scale bar: 50 nm. **(F)** Particle size experiments showing the diameter of extracted exosomes. **(G)** Representative images of exosome localization in cells. Scale bar: 10 μm.

Data were assessed with unpaired Student’s *t* test and presented as mean±SD. * *P* < 0.05; ** *P*< 0.01; *** *P* < 0.001; ns, no significance.

**
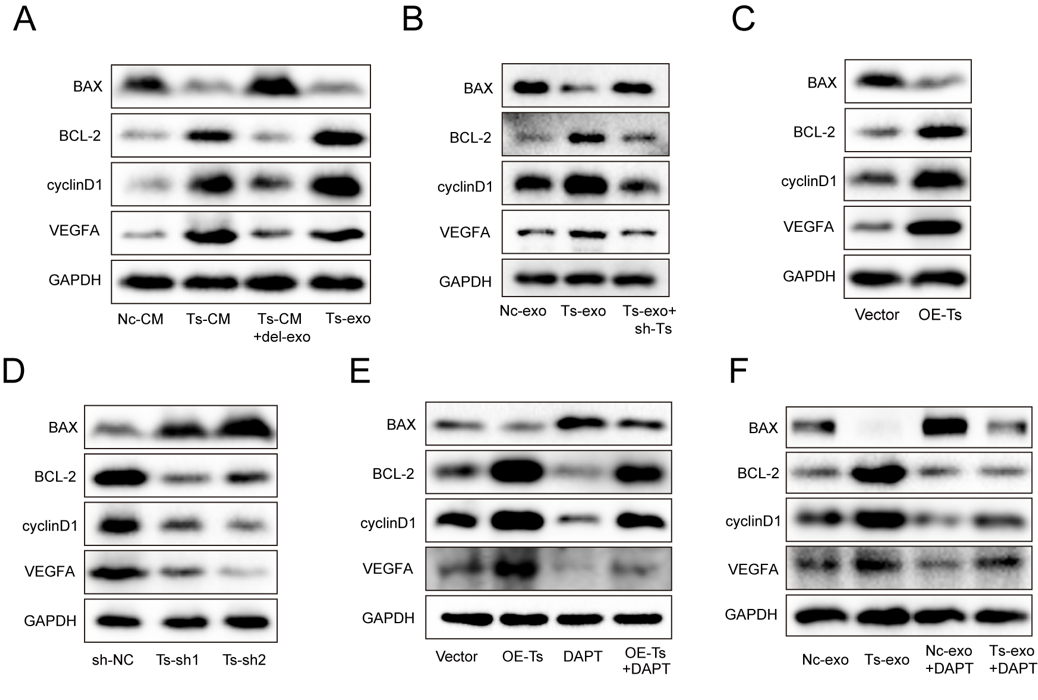
**

**Figure S5 Exosomes transport TRPM2-AS to HUVECs and increased the expression of tumour angiogenesis-related proteins.**

**(A-B)** Western blot assays showing the protein expression level of BAX, BCL-2, cyclinD1, and VEGFA in HUVECs cultured in Nc-CM/Ts-CM/Ts-CM+del-exo/Ts-exo **(A)**, Nc-exo/Ts-exo/Ts-exo+sh-Ts **(B)**. Nc-CM: conditioned medium from NOZ cells overexpressing empty plasmid. Ts-CM: conditioned medium from NOZ cells overexpressing TRPM2-AS. Ts-CM+del-exo: culture medium from NOZ cells overexpressing TRPM2-AS after exclusion of exosomes. Ts-exo: exosomes from NOZ cells overexpressing TRPM2-AS. Nc-exo: exosomes from NOZ cells overexpressing empty plasmid. Ts-exo+sh-Ts: knockdown TRPM2-AS using shRNA after HUVECs were co-cultured with Ts-exo. **(C-D)** Western blot assays showing the protein expression level of BAX, BCL-2, cyclinD1, and VEGFA in HUVECs with overexpression **(C)** and knockdown **(D)** of TRPM2-AS. (**E-F)** Western blot validation of BAX, BCL-2, cyclinD1, and VEGFA in TRPM2-AS overexpressing/control HUVECs with/without DAPT treatment **(E)**, and HUVECs cultured in Nc-exo/Ts-exo with/without DAPT treatment **(F)**.

**
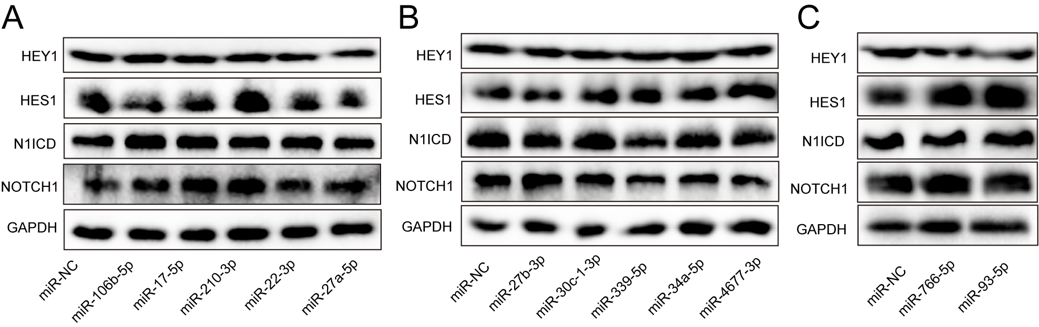
**

**Figure S6 TRPM2-AS activates NOTCH1 signaling pathway in independent of the sponge miRNA function.**

**(A-C)** After transfection of the miR-NC, miR-106b-5p, miR-17-5p, miR-210-3p, miR-22-3p, miR-27a-5p, miR-27b-3p, miR-30c-1-3p, miR-339-5p, miR-34a-5p, miR-4677-3p, miR-766-5p and miR-93-5p mimic into HUVECs, the activation of NOTCH1 signaling pathway was detected by western blot.

**
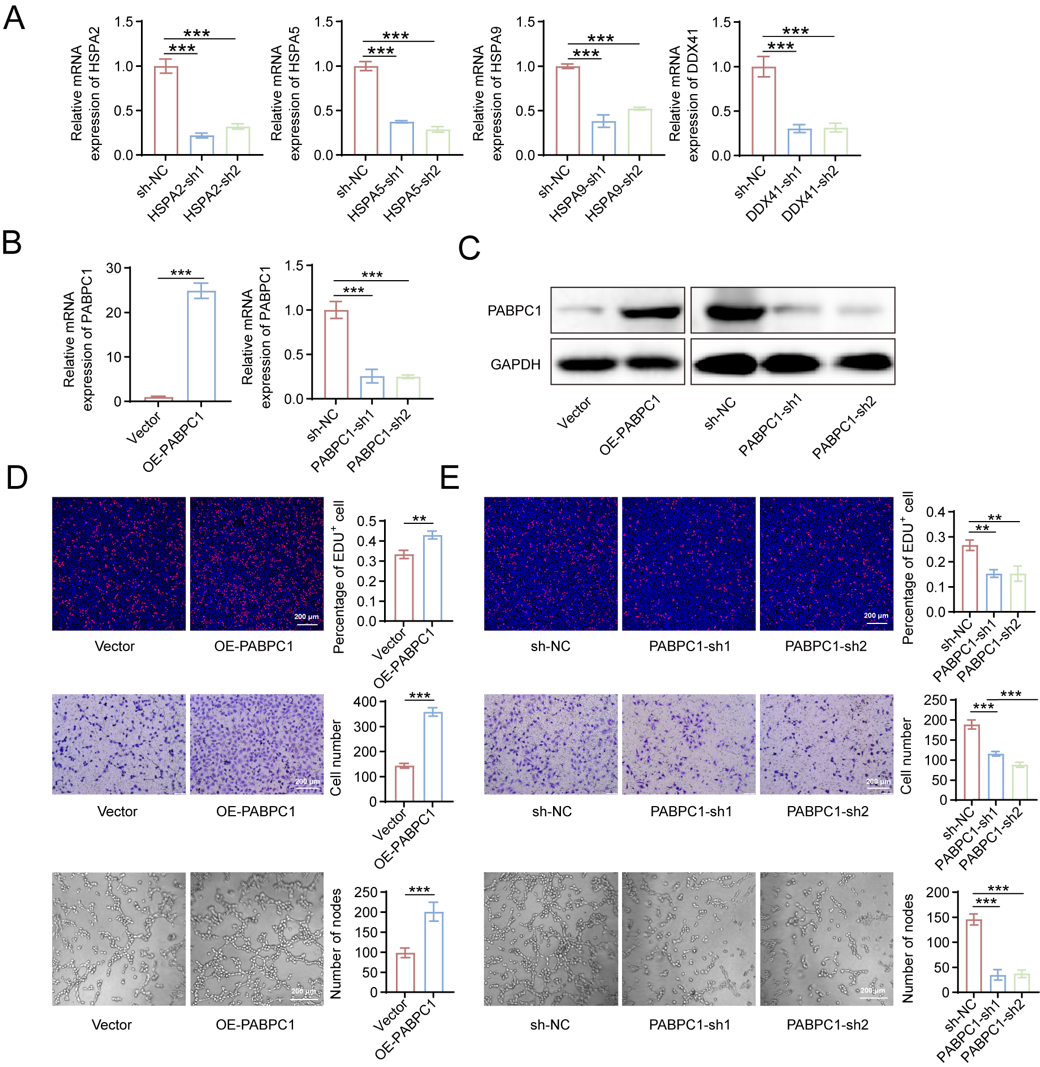
**

**Figure S7 PABPC1 facilitates tumor angiogenesis.**

**(A)** The knockdown efficiency of HSPA2, HSPA5, HSPA9, and DDX41was evaluated by RT-qPCR. **(B-C)** RT-qPCR **(B)** and western blot **(C)** validation of the construction of stable PABPC1 overexpression/knockdown HUVECs by lentivirus transfection. **(D-E)** EDU, transwell, and tube formation assays to verify the angiogenic ability of HUVECs with PABPC1 overexpression **(D)** and knockdown **(E)**. Scale bar: 200 μm.

Data were assessed with unpaired Student’s *t* test or one-way ANOVA and presented as mean±SD. ** *P*< 0.01; *** *P* < 0.001.

**
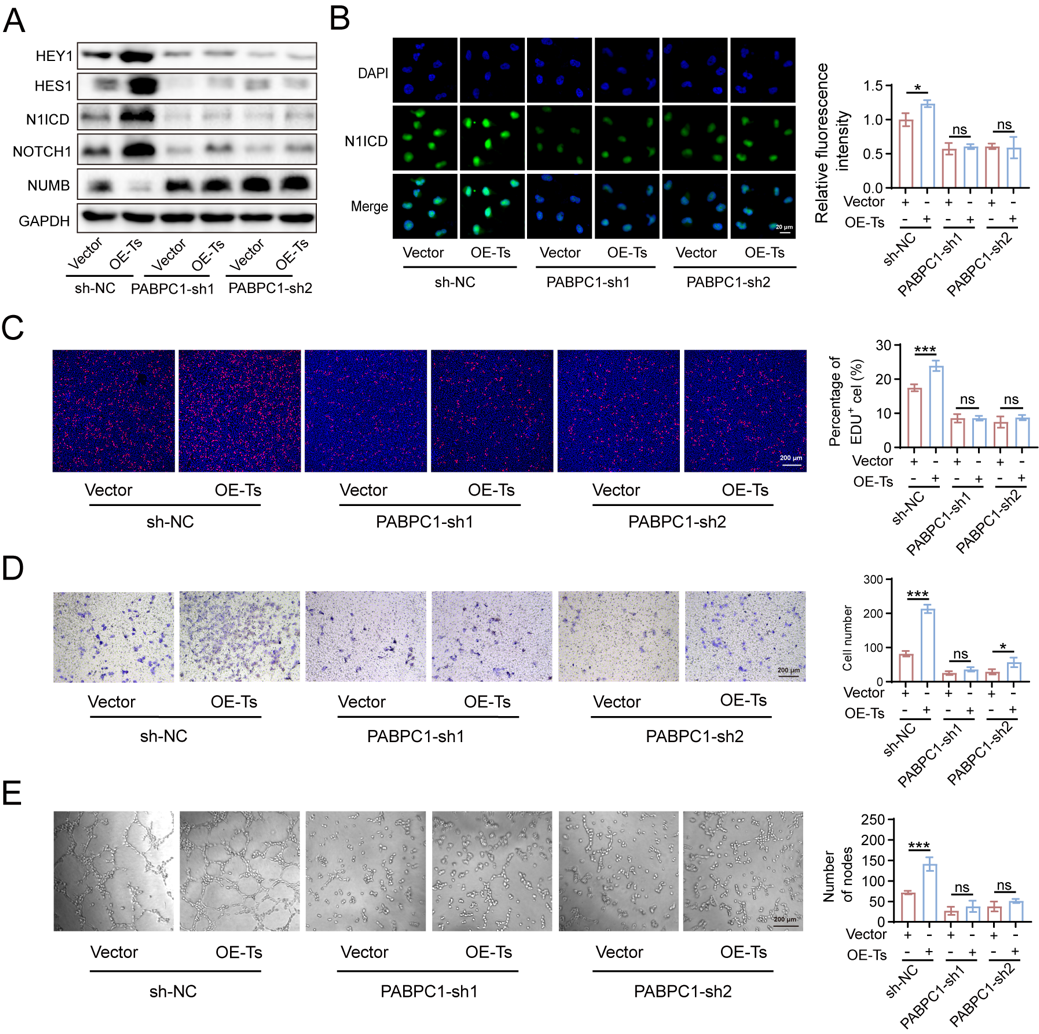
**

**Figure S8 The inhibitory effects of TRPM2-AS on NOTCH1 signaling pathway and GBC angiogenesis depend on PABPC1.**

**(A)** Western blot showing the protein expression of HEY1, HES1, N1ICD, NOTCH1, and NUMB in the control group and in the TRPM2-AS overexpression group with/without PABPC1 knockdown. **(B)** Representative immunofluorescence images of N1ICD expression in the control group and in the TRPM2-AS overexpression group with/without PABPC1 knockdown. Green fluorescence: primary antibody stained N1ICD; blue fluorescence: DAPI-stained nuclei. Scale bar: 20 μm. **(C-E)** EDU **(C)**, transwell **(D)**, and tube formation assays **(E)** to verify the angiogenic ability of HUVECs in the control group and in the TRPM2-AS overexpression group with/without PABPC1 knockdown. Scale bar: 200 μm.

Data were assessed with one-way ANOVA and presented as mean ± SD. * *P* < 0.05; *** *P* < 0.001; ns, no significance.

**
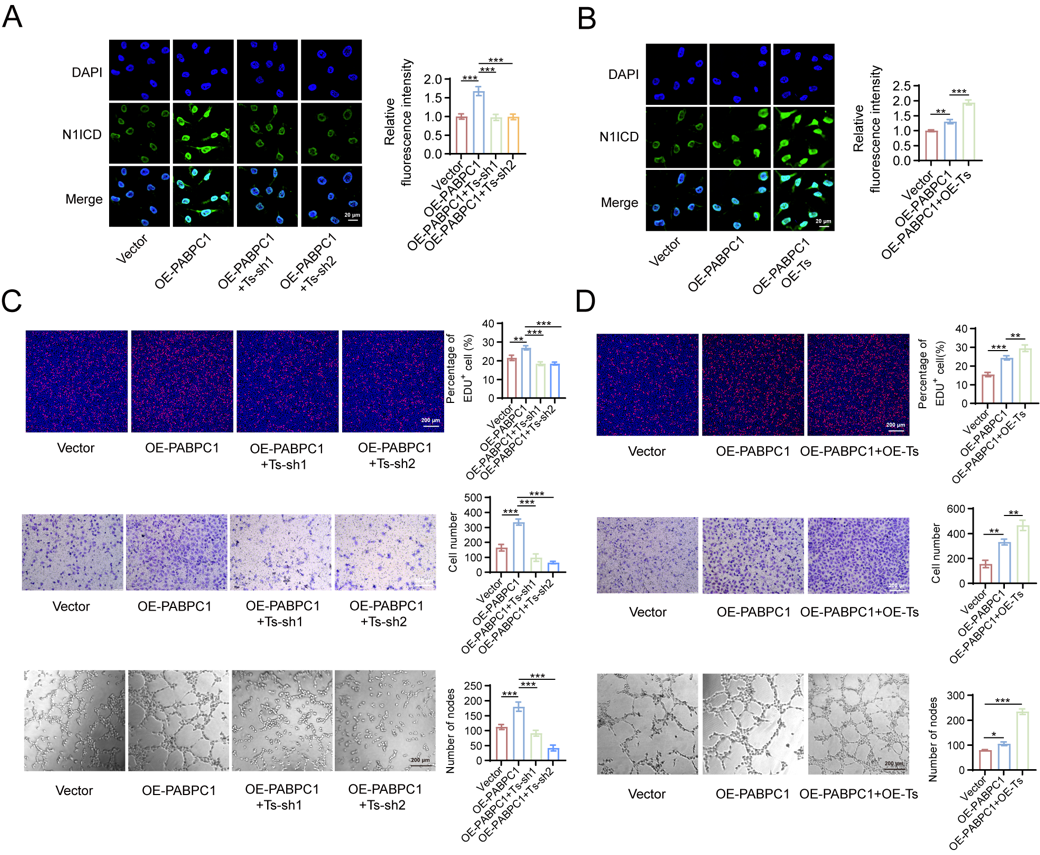
**

**Figure S9 TRPM2-AS enhances the PABPC1 activation effects on NOTCH1 signaling pathway and promotion on tumor angiogenesis.**

**(A-B)** Representative immunofluorescence images of N1ICD expression in the control group and in the PABPC1 overexpression group with/without TRPM2-AS knockdown **(A)** and with/without TRPM2-AS overexpression **(B).** Green fluorescence: primary antibody stained N1ICD; blue fluorescence: DAPI-stained nuclei. Scale bar: 20 μm. **(C-D)** EDU, transwell, and tube formation assays were used to verify the angiogenic capacity of HUVECs in the control and PABPC1 overexpression groups with/without TRPM2-AS knockdown **(C)** and with/without TRPM2-AS overexpression **(D)**. Scale bar: 200 μm.

Data were assessed with one-way ANOVA and presented as mean ± SD. * *P* < 0.05; ** *P* < 0.01; *** *P* < 0.001.

**
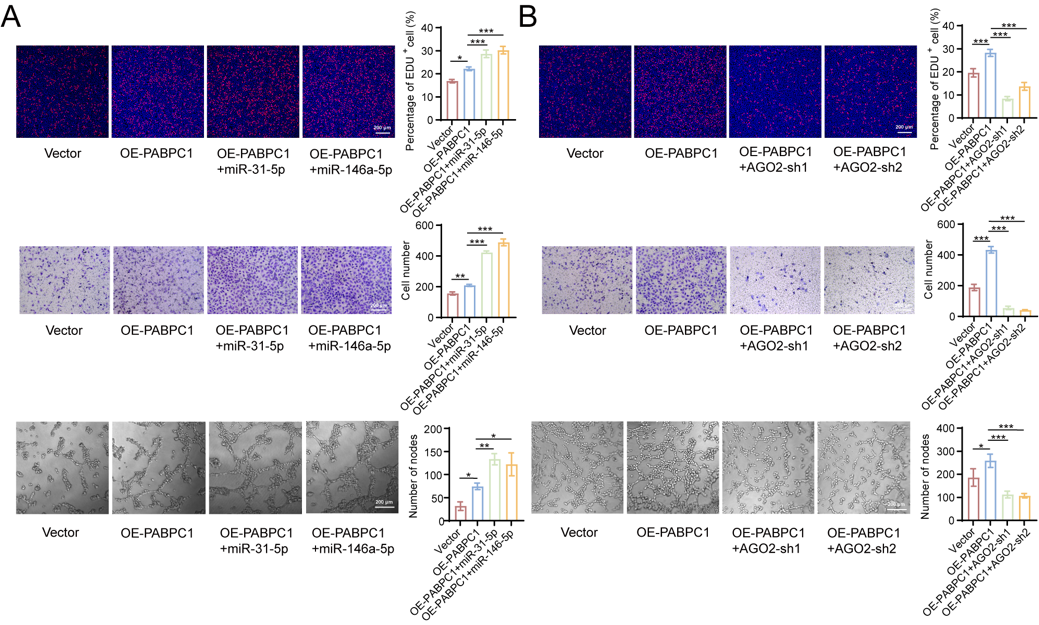
**

**Figure S10 PABPC1 promotes tumours angiogenesis by enhancing miRNA-mediated NUMB degradation.**

**(A-B)** EDU, transwell, and tube formation assays verified the angiogenesis abilities of HUVECs in the control and PABPC1 overexpression groups with/without overexpression of miR-31-5p/miR-146a-5p **(A)** and knockdown of AGO2 **(B)**. Scale bar: 200 μm.

Data were assessed with one-way ANOVA and presented as mean±SD. * *P* < 0.05; ** *P* < 0.01; *** *P* < 0.001.

**Supplementary Tables**

**Supplementary Table S1: List of the RNAi targeted sequence.**

| **Gene name** | **Sequences** |
| --- | --- |
| TRPM2-AS shRNA-1 | 5’-CGAACCUUCCCUAAUAGAA-3’ |
| TRPM2-AS shRNA-2 | 5’-GGUCGAACACCAGCUCUGA-3’ |
| IGF2BP2 shRNA-1 | 5’-AGAUAGAGAUUAUGAAGAA-3’ |
| IGF2BP2 shRNA-2 | 5’-GUUGAUUACUCAGUCUCUA-3’ |
| PABPC1 shRNA-1 | 5’-GCAUGAAGAUGCACAGAAA-3’ |
| PABPC1 shRNA-2 | 5’-AGGCGAUGCUCUACGAGAA-3’ |
| HSPA2 shRNA-1 | 5’-GAGGCUGAUUGGACGGAAA-3’ |
| HSPA2 shRNA-2 | 5’-AAGUGCAAGUAGAGUACAA-3’ |
| HSPA5 shRNA-1 | 5’-GCGUCGGCGUGUUCAAGAA-3’ |
| HSPA5 shRNA-2 | 5’-GCAUCAAGCAAGAAUUGAA-3’ |
| HSPA9 shRNA-1 | 5’-AGACAAAUCAGAAGACAAA-3’ |
| HSPA9 shRNA-2 | 5’-GCGAUAUGAUGAUCCUGAA-3’ |
| DDX41 shRNA-1 | 5’-GAGCAUUGAUGUCAGUGAA-3’ |
| DDX41 shRNA-2 | 5’-GCAGGACAGCGGUAGUGAA-3’ |
| AGO2 shRNA-1 | 5’-GCAAGAAGAGAUUAGCAAA-3’ |
| AGO2 shRNA-2 | 5’-GGAGAACAAUCAAAUUACA-3’ |
| sh-NC | 5’-CCTAAGGTTAAGTCGCCCTCG-3’ |

**Supplementary Table S2: List of the miRNA and their sequence.**

| **miRNA name** | **Sequences** |
| --- | --- |
| miR-NC | UUUGUACUACACAAAAGUACUG |
| miR-106b-5p | UAAAGUGCUGACAGUGCAGAU |
| miR-17-5p | CAAAGUGCUUACAGUGCAGGUAG |
| miR-210-3p | CUGUGCGUGUGACAGCGGCUGA |
| miR-22-3p | AAGCUGCCAGUUGAAGAACUGU |
| miR-27a-5p | AGGGCUUAGCUGCUUGUGAGCA |
| miR-27b-3p | UUCACAGUGGCUAAGUUCUGC |
| miR-30c-1-3p | CUGGGAGAGGGUUGUUUACUCC |
| miR-339-5p | UCCCUGUCCUCCAGGAGCUCACG |
| miR-34a-5p | UGGCAGUGUCUUAGCUGGUUGU |
| miR-4677-3p | UCUGUGAGACCAAAGAACUACU |
| miR-766-5p | AGGAGGAAUUGGUGCUGGUCUU |
| miR-93-5p | CAAAGUGCUGUUCGUGCAGGUAG |
| miR-31-5p | AGGCAAGAUGCUGGCAUAGCU |
| miR-146a-5p | UGAGAACUGAAUUCCAUGGGUU |

**Supplementary Table S3: List of the primers and their sequence.**

| **Primer name** | **Sequences** |
| --- | --- |
| TRPM2-AS forward | CTTTTGGAACTGGCTGAGGTC |
| TRPM2-AS reverse | GGATGAGTAAGTGGCTGGTGG |
| GAPDH forward | GGAGTCCACTGGCGTCTTCA |
| GAPDH reverse | GTCATGAGTCCTTCCACGATACC |
| NUMB forward | GCAGCAGACATTCCCTCACT |
| NUMB reverse | AGAACCGTTGAGGTGCTGAG |
| U6 forward | TGCTTCGGCAGCACATATAC |
| U6 reverse | TCACGAATTTGCGTGTCATC |
| β-actin forward | GGGAAATCGTGCGTGACATTAAG |
| β-actin reverse | TGTGTTGGCGTACAGGTCTTTG |
| IGF2BP2 forward | AAGCTAAGCGGGCATCAGTT |
| IGF2BP2 reverse | CGCAGCGGGAAATCAATCTG |
| m6A peak 1 forward | GCAATGAGCTGAACTCGCAG |
| m6A peak 1 reverse | TGTGTCTGAACCTGGTCACG |
| m6A peak 2 forward | ACTCTGCAGTGCGGTATGTG |
| m6A peak 2 reverse | CTCTCTCCTGGGACCGACA |
| m6A peak 3 forward | ACCCCCTTTTCTTCCACCAG |
| m6A peak 3 reverse | AGCTTTTATTCTAGGCCAAC |
| LINC01823 forward | TGCTAGCTAGTCAAAGGATAGAAA |
| LINC01823 reverse | CATCACAGGTGGCAAAAGCC |
| EPHA1-AS1 forward | CCCATGGTGAAGCATCAGGT |
| EPHA1-AS1 reverse | GAGAGGATCTGCCCGAATGG |
| APCDD1L forward | GAGCCTTGGAAAGGAGGACC |
| APCDD1L reverse | GATCCATGCAGGTGGGAACA |
| MEF2C-AS1 forward | GCCTGGCCTTCATTTTCAGC |
| MEF2C-AS1 reverse | GAGGGGTCCTGAGAGGTTCA |
| LINC02039 forward | GGAGGCCCACATGTGATGAA |
| LINC02039 reverse | TGGAGGCGAGTCCCAGAATA |
| LINC00261 forward | AGGATTCTGCATGTGGGTGG |
| LINC00261 reverse | GGTGAGCCCCAGCCTTATTT |
| ASB16-AS1 forward | GCCCTGAGGCAAACATACCT |
| ASB16-AS1 reverse | CATGTGGGTGAGGCAAGACT |
| KIF1C-AS1 forward | CTTGCAAACTGCCACCTGAC |
| KIF1C-AS1 reverse | GATACCAGGGTTCCTCTGCG |
| ANKRD44-IT1 forward | TCTGACTGGTCTCCTTCCGT |
| ANKRD44-IT1 reverse | ACACCCCTCGATTGCTTGAA |
| HSPA2 forward | GTTCAAGCGCAAGCACAAGA |
| HSPA2 reverse | CACGATCTCCTGGATCTGGC |
| HSPA5 forward | TCTTGTTGGTGGCTCGACTC |
| HSPA5 reverse | GGTCATGACACCTCCCACAG |
| HSPA9 forward | AGAAGACCGGCGAAAGAAGG |
| HSPA9 reverse | GCCAGGAGCTCCCTCATTTT |
| DDX41 forward | GGCAGCTACTGCTCCAGAA |
| DDX41 reverse | TAAGGTGCTGGTGCTGATCC |

**Supplementary Table S4:** **List of the antibodies and their manufacturer’s information**

| **Primer names** | **Company** | **Number** |
| --- | --- | --- |
| IGF2BP1 | Proteintech | 22803-1-AP |
| IGF2BP2 | Proteintech | 11601-1-AP |
| IGF2BP3 | Proteintech | 14642-1-AP |
| YTHDF1 | Proteintech | 17479-1-AP |
| YTHDF2 | Proteintech | 24744-1-AP |
| YTHDF3 | Proteintech | 25537-1-AP |
| NOTCH1 | Cell Signaling | 3608 |
| NICD | Cell Signaling | 4147 |
| HES1 | HUABIO | ET1610-97 |
| HEY1 | Proteintech | 19929-1-AP |
| NUMB | HUABIO | ET1703-02 |
| PABPC1 | Proteintech | 10970-1-AP |
| PABPC1 | Proteintech | 66809-1-Ig |
| Myc-tag | Proteintech | 60003-2-Ig |
| Myc-tag | Cell Signaling | 3724 |
| AGO2 | Proteintech | 67934-1-Ig |
| AGO2 | Abcam | ab186733 |
| CD34 | HUABIO | ET1606-1 |
| CD9 | Proteintech | 60232-1-Ig |
| CD63 | Proteintech | 67605-1-Ig |
| CD81 | Proteintech | 66866-1-Ig |

**Supplementary Table S5: Comparison of baseline characteristics and clinicopathological features in patients with high or low TRPM2-AS expression.**

| **Variables** | **High expression of TRPM2-AS** | **Low expression**  **of TRPM2-AS** | ***P*** | **OR** | **95% CI** |
| --- | --- | --- | --- | --- | --- |
| **Age**  **(>60/≤60)** | 16/14 | 13/17 | 0.4383 | 1.495 | 0.5132 to 3.927 |
| **Gender**  **(Female/Male)** | 23/7 | 26/4 | 0.3169 | 0.5055 | 0.1518 to 1.989 |
| **Tumor size**  **(>5 cm/≤5 cm)** | 20/10 | 10/20 | 0.0098 | 4 | 1.303 to 10.97 |
| **Lymph node**  **(Postive/Negative)** | 11/19 | 4/26 | 0.0369 | 3.763 | 1.000 to 11.82 |
| **Liver involvement**  **(Yes/No)** | 9/21 | 2/28 | 0.0195 | 6 | 1.250 to 29.18 |
| **Differentiation**  **(Poor/Well+moderate)** | 16/14 | 6/24 | 0.0074 | 4.571 | 1.532 to 15.02 |
| **CA19-9**  **(>50 U/ml /≤50 U/ml)** | 23/7 | 10/20 | 0.0007 | 6.571 | 1.962 to 20.51 |
| **TNM stages**  **(III+IV/I+II)** | 18/12 | 8/22 | 0.0092 | 4.125 | 1.309 to 11.19 |
| **Jaundice**  **(Yes/No)** | 3/27 | 7/23 | 0.1659 | 0.3651 | 0.09568 to 1.637 |
| **Radical operation**  **(Yes/No)** | 27/3 | 23/7 | 0.1659 | 2.739 | 0.6108 to 10.45 |
| **CEA**  **(>3** **ng/mL/≤3** **ng/mL)** | 14/16 | 17/13 | 0.4383 | 0.6691 | 0.2546 to 1.949 |
| **ALP**  **(>80** **U/L/≤80 U/L)** | 7/23 | 5/25 | 0.5186 | 1.522 | 0.4220 to 5.071 |

**Supplementary Table S6:** **Univariate and multivariate cox-regression analysis to overall survival in patients with high/low TRPM2-AS expression.**

| **Variables** | **Univariate** | | | **Multivariate** | | |
| --- | --- | --- | --- | --- | --- | --- |
|  | **HR** | **95% CI** | ***P*** | **HR** | **95% CI** | ***P*** |
| **Tumor size**  **(>5 cm/≤5 cm)** | 1.05 | 0.564-1.954 | 0.878 | 0.85 | 0.434-1.668 | 0.637 |
| **Lymph node**  **(Postive/Negative)** | 1.121 | 0.602-2.087 | 0.719 | 0.865 | 0.441-1.697 | 0.673 |
| **Liver involvement**  **(Yes/No)** | 0.696 | 0.371-1.307 | 0.26 | 2.549 | 1.177-5.521 | 0.018 |
| **Differentiation**  **(Poor/Well+moderate)** | 3.504 | 1.844-6.658 | ＜0.0001 | 3.612 | 1.5-6.665 | 0.002 |
| **CA19-9**  **(>50 U/ml /≤50 U/ml)** | 1.507 | 0.803-2.831 | 0.202 | 0.999 | 0.481-2.074 | 0.998 |
| **TNM stages**  **(III+IV/I+II)** | 2.363 | 1.261-4.425 | 0.007 | 2.722 | 1.265-5.855 | 0.01 |
| **TRPM2-AS expression (High/Low)** | 2.401 | 1.256-4.587 | 0.008 | 2.43 | 1.182-4.998 | 0.016 |

**Supplementary Table S7: Univariate and multivariate cox-regression analysis to recurence-free survival in patients with high/low TRPM2-AS expression.**

| **Variables** | **Univariate** | | | **Multivariate** | | |
| --- | --- | --- | --- | --- | --- | --- |
|  | **HR** | **95% CI** | ***P*** | **HR** | **95% CI** | ***P*** |
| **Tumor size**  **(>5 cm/≤5 cm)** | 0.93 | 0.52-1.664 | 0.808 | 0.786 | 0.421-1.469 | 0.451 |
| **Lymph node**  **(Postive/Negative)** | 1.255 | 0.702-2.245 | 0.444 | 0.978 | 0.514-1.862 | 0.947 |
| **Liver involvement**  **(Yes/No)** | 1.496 | 0.829-2.7 | 0.181 | 2.761 | 1.35-5.646 | 0.005 |
| **Differentiation**  **(Poor/Well+moderate)** | 3.806 | 2.015-7.188 | <0.0001 | 3.279 | 1.548-6.946 | 0.002 |
| **CA19-9**  **(>50 U/ml /≤50 U/ml)** | 1.438 | 0.799-2.586 | 0.225 | 1.037 | 0.52-2.066 | 0.918 |
| **TNM stages**  **(III+IV/I+II)** | 2.396 | 1.328-4.323 | 0.004 | 2.798 | 1.331-5.883 | 0.007 |
| **TRPM2-AS expression**  **(High/Low)** | 2.065 | 1.143-3.732 | 0.016 | 2.124 | 1.122-4.021 | 0.021 |

**Supplementary Table S8: Univariate and multivariate cox-regression analysis to overall survival in patients with high/low IGF2BP2 expression.**

| **Variables** | **Univariate** | | | **Multivariate** | | |
| --- | --- | --- | --- | --- | --- | --- |
|  | **HR** | **95% CI** | ***P*** | **HR** | **95% CI** | ***P*** |
| **Tumor size**  **(>5 cm/≤5 cm)** | 0.7858 | 0.4461 to 1.384 | 0.3918 | 0.491 | 0.263 to 0.914 | 0.025 |
| **Lymph node**  **(Postive/Negative)** | 0.9213 | 0.5229 to 1.623 | 0.7711 | 0.920 | 0.476 to 1.778 | 0.805 |
| **Liver involvement**  **(Yes/No)** | 2.51 | 1.391 to 4.528 | 0.0008 | 0.824 | 0.345 to 1.970 | 0.664 |
| **Differentiation**  **(Poor/Well+moderate)** | 2.77 | 1.522 to 5.043 | 0.0002 | 2.405 | 1.190 to 4.861 | 0.014 |
| **CA19-9**  **(>50 U/ml /≤50 U/ml)** | 1.227 | 0.6961 to 2.163 | 0.4671 | 1.236 | 0.637 to 2.400 | 0.531 |
| **TNM stages**  **(III+IV/I+II)** | 2.804 | 1.551 to 5.070 | 0.0002 | 3.794 | 1.688 to 8.529 | 0.001 |
| **IGF2BP2 expression**  **(High/Low)** | 3.367 | 1.862 to 6.090 | <0.0001 | 2.647 | 1.280 to 5.474 | 0.009 |

**Supplementary Table S9: Univariate and multivariate cox-regression analysis to recurence-free survival in patients with high/low IGF2BP2 expression.**

| **Variables** | **Univariate** | | | **Multivariate** | | |
| --- | --- | --- | --- | --- | --- | --- |
|  | **HR** | **95% CI** | ***P*** | **HR** | **95% CI** | ***P*** |
| **Tumor size**  **(>5 cm/≤5 cm)** | 0.8498 | 0.5051 to 1.430 | 0.5235 | 0.594 | 0.341 to 1.034 | 0.066 |
| **Lymph node**  **(Postive/Negative)** | 1.151 | 0.6847 to 1.934 | 0.5828 | 1.060 | 0.592 to 1.895 | 0.845 |
| **Liver involvement**  **(Yes/No)** | 1.684 | 0.9868 to 2.875 | 0.0371 | 0.716 | 0.306 to 1.674 | 0.440 |
| **Differentiation**  **(Poor/Well+moderate)** | 3.011 | 1.721 to 5.269 | <0.0001 | 2.843 | 1.427 to 5.664 | 0.003 |
| **CA19-9**  **(>50U/ml/≤50 U/ml)** | 1.169 | 0.6947 to 1.966 | 0.5407 | 1.015 | 0.547 to 1.886 | 0.961 |
| **TNM stages**  **(III+IV/I+II)** | 2.394 | 1.388 to 4.129 | 0.0003 | 1.826 | 0.948 to 3.516 | 0.072 |
| **IGF2BP2 expression**  **(High/Low)** | 2.711 | 1.565 to 4.697 | <0.0001 | 2.971 | 1.383 to 6.386 | 0.005 |
